# Pemafibrate suppresses NLRP3 inflammasome activation in the liver and heart in a novel mouse model of steatohepatitis-related cardiomyopathy

**DOI:** 10.1038/s41598-022-06542-8

**Published:** 2022-02-22

**Authors:** Kotaro Kanno, Masahiro Koseki, Jiuyang Chang, Ayami Saga, Hiroyasu Inui, Takeshi Okada, Katsunao Tanaka, Masumi Asaji, Yinghong Zhu, Seiko Ide, Shigeyoshi Saito, Tomoaki Higo, Daisuke Okuzaki, Tohru Ohama, Makoto Nishida, Yoshihiro Kamada, Masafumi Ono, Toshiji Saibara, Shizuya Yamashita, Yasushi Sakata

**Affiliations:** 1 Division of Cardiovascular Medicine, Department of Medicine, Osaka University Graduate School of Medicine, 2-2-B5 Yamadaoka, Suita, Osaka, 565-0871 Japan; 2 Health Care Division, Health and Counselling Centre, Osaka University, Osaka, Japan; 3 Division of Health Sciences, Department of Medical Physics and Engineering, Osaka University Graduate School of Medicine, Osaka, Japan; 4 Genome Information Research Centre, Research Institute for Microbial Diseases, Osaka University, Osaka, Japan; 5 Department of Dental Anaesthesiology, Osaka University Graduate School of Dentistry, Osaka, Japan; 6 Department of Advanced Metabolic Hepatology, Osaka University Graduate School of Medicine, Osaka, Japan; 7 Division of Innovative Medicine for Hepatobiliary and Pancreatology, Faculty of Medicine, Kagawa University, Kagawa, Japan; 8 Department of Gastroenterology and Hepatology, Kochi Medical School, Kochi, Japan; 9 Department of Cardiology, Rinku General Medical Centre, Osaka, Japan

**Keywords:** Cardiology, Gastroenterology, Molecular medicine, Risk factors

## Abstract

Although patients with nonalcoholic fatty liver disease have been reported to have cardiac dysfunction, and appropriate model has not been reported. We established a novel mouse model of diet-induced steatohepatitis-related cardiomyopathy and evaluated the effect of pemafibrate. C57Bl/6 male mice were fed a (1) chow diet (C), (2) high-fat, high-cholesterol, high-sucrose, bile acid diet (NASH diet; N), or (3) N with pemafibrate 0.1 mg/kg (NP) for 8 weeks. In the liver, macrophage infiltration and fibrosis in the liver was observed in the N group compared to the C group, suggesting steatohepatitis. Free cholesterol accumulated, and cholesterol crystals were observed. In the heart, free cholesterol similarly accumulated and concentric hypertrophy was observed. Ultrahigh magnetic field magnetic resonance imaging revealed that the left ventricular (LV) ejection fraction (EF) was attenuated and LV strain was focally impaired. RNA sequencing demonstrated that the NOD-like receptor and PI3 kinase-Akt pathways were enhanced. mRNA and protein expression of inflammasome-related genes, such as Caspase-1, NLRP3, and IL-1β, were upregulated in both the liver and heart. In the NP compared to the N group, steatohepatitis, hepatic steatosis, and cardiac dysfunction were suppressed. Sequential administration of pemafibrate after the development of steatohepatitis-related cardiomyopathy recovered hepatic fibrosis and cardiac dysfunction.

## Introduction

Reports have indicated that the number of patients with nonalcoholic fatty liver disease (NAFLD) is markedly increasing worldwide^1^. Recent studies have demonstrated that patients with NAFLD, including nonalcoholic fatty liver (NAFL) and nonalcoholic steatohepatitis (NASH), have an increased risk of cardiovascular diseases^2–4^. In fact, NAFLD and atherosclerotic cardiovascular diseases share similar metabolic backgrounds, such as obesity, diabetes, hypertriglyceridemia, and hypo-high-density lipoprotein cholesterol (HDL-C)^5,6^. More importantly, recent clinical studies have suggested that the presence of NAFLD may induce cardiac dysfunction. Lee et al. reported that the presence of NAFLD and the degree of hepatic fibrosis were associated with echocardiographic early diastolic LV filling velocity/peak atrial filling velocity (E/A) ratio and ratio between the E wave velocity from mitral inflow and the E' velocity (E/e'), which indicate left ventricular (LV) diastolic function^7^. Canada et al. also reported that peak VO2 decreased with increasing fibrosis stage^8^. However, no mouse models of simultaneous steatohepatitis and cardiomyopathy have been reported.

Over the last thirty years, the mortality of cardiovascular diseases has decreased because of the use of statins^9^; however, there are a certain number of patients with atherosclerotic cardiovascular diseases. Therefore, residual risks must be monitored, such as remnant cholesterol, and therapies in addition to statins must be developed^10^. A decade ago, the ACCORD lipid trial investigated whether coadministration of a statin and fenofibrate might reduce cardiovascular events; however, this approach did not achieve significant risk reduction except in some individuals with high triglycerides and low HDL-C^11^. Pemafibrate is a novel selective peroxisome proliferator-activated receptor α (PPARα) modulator that has recently been developed in Japan, and it decreases serum TG levels and increases HDL-C levels^12,13^. Previous clinical trials have shown its efficacy and safety, even when used in combination with statins^14–16^. Therefore, coadministration of pemafibrate with statins would be expected to reduce the residual risk of cardiovascular outcomes in patients with diabetes. The phase 3 clinical trial PROMINENT study is currently ongoing, and it includes approximately 10,000 participants with type 2 diabetes, mild-to-moderate hypertriglyceridemia (TG: 200–499 mg/dl) and low HDL-C levels (HDL-C: ≤ 40 mg/dl)^17^. In animal models, pemafibrate has demonstrated various effects on systemic organs. In a diet-induced obesity model, adipose tissue weight was reduced^18^. Honda et al. revealed that pemafibrate attenuated hepatic triglyceride contents, macrophage infiltration, and hepatic fibrosis in a diet-induced steatohepatitis model^19^. Yoshida et al. also reported that pemafibrate and statins improved vascular endothelial dysfunction in dahl/salt-sensitive rats^20^. Although a previous report indicated that another fibrate, fenofibrate, had a protective role against cardiac injury in a porcine myosin-induced rat myocarditis model^21^, no study has investigated whether pemafibrate might regulate cardiac inflammation or cholesterol metabolism in the heart.

Therefore, we decided to evaluate a novel model of steatohepatitis accompanied by cardiac dysfunction by a cholesterol-rich atherogenic diet (high fat, high cholesterol, and bile salt diet) and elucidate the underlying molecular mechanism. We also tested the effect of pemafibrate on cholesterol metabolism in tissues, hepatic fibrosis, and cardiac dysfunction as a potential therapeutic agent for steatohepatitis and related diseases.

## Results

### High-fat/high-cholesterol/high-sucrose/bile acid diet induces severe inflammation and fibrosis in the liver, which are suppressed by pemafibrate

**Figure 1:**
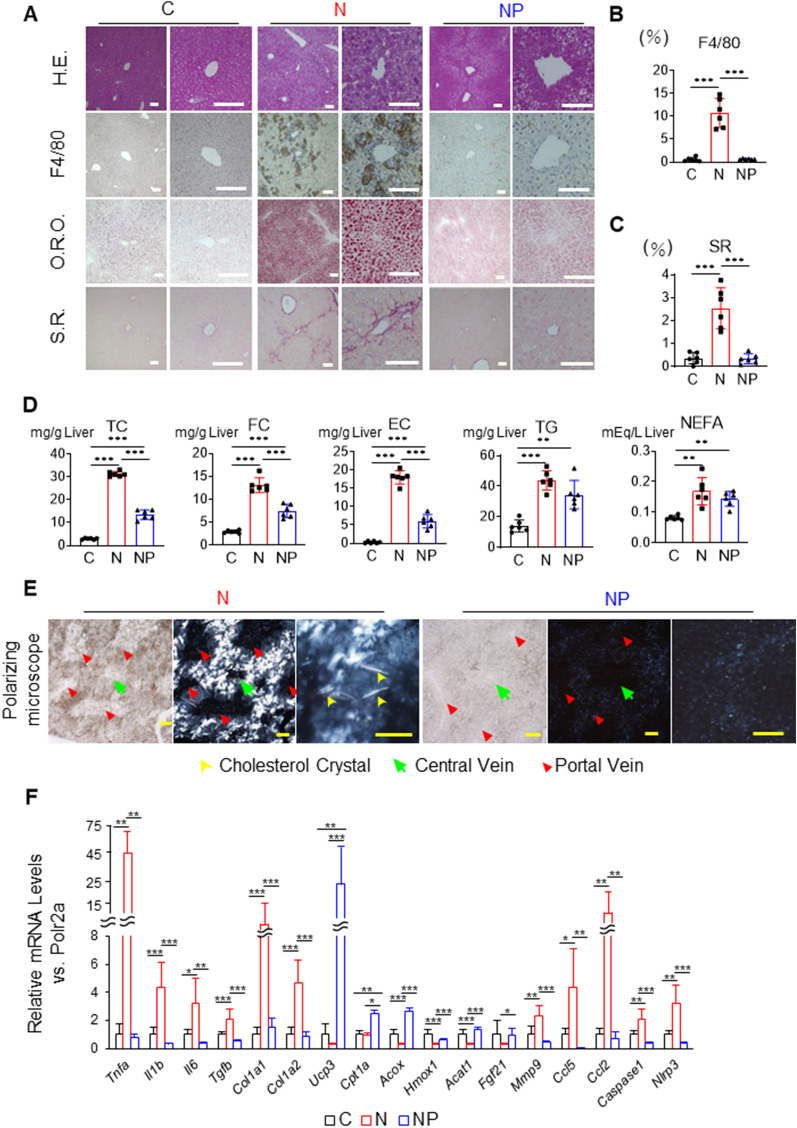
Effects of the NASH diet and pemafibrate on the liver of wild-type male mice. (**A**) Histological examinations of samples from mice in the chow diet-fed group (C group), NASH diet-fed group (N group), and NASH diet + pemafibrate-fed group (NP group). (**B**) Percentage of examined area positive for F4/80. (**C**) Percentage of examined area positive for Sirius red. (**D**) Hepatic lipid contents by lipid extraction analysis. (**E**) Polarized microscope images of cholesterol crystals. The yellow, green, and red arrows indicate cholesterol crystals, central vein, and portal vein, respectively. (**F**) mRNA expression associated with inflammation, fibrosis, β-oxidation, chemokines, and inflammasomes. The mean value of the expression of the C group, obtained by correcting each CT value with the respective housekeeping gene, was used as a control (one) to indicate the relative expression. Scale bar: 100 µm. The results are presented as the mean ± SD, and p values were calculated using one-way ANOVA with Tukey’s post-hoc test. *P < 0.05, **P < 0.01 and ***P < 0.001, C vs. N group or C vs. NP group or N vs. NP group. The number of mice in each group: C, n = 6; N, n = 6; NP, n = 6. H.E., haematoxylin–eosin; O.R.O., oil red O; S.R., Sirius red; TC, total cholesterol; FC, free cholesterol; EC, esterified cholesterol; TG, triglycerides; NEFA, nonesterified fatty acids.

Histological images of the livers of mice fed the C, N, and NP diets were examined. In the N group, the infiltration of inflammatory cells and F4/80-positive macrophages was more pronounced than that in the C group (10.7 ± 3.2 vs. 0.55 ± 0.44%, *P* < 0.001, Fig. 1A,B). SR staining-positive fibrosis areas were also increased (2.5 ± 0.9 vs. 0.35 ± 0.25%, *P* < 0.001, Fig. 1A,C). In addition, O.R.O staining showed that the area of lipid droplets was increased (Fig. 1A). The NAFLD activity score (NAS) was 7.3 ± 0.8, and the fibrosis stage was 3.2 ± 0.4 (Table 1). On the other hand, in the NP group compared to the N group, inflammatory cells and F4/80-positive macrophages were significantly suppressed (0.62 ± 0.10 vs. 10.7 ± 3.2%, *P* < 0.001, Fig. 1A,B). Importantly, SR staining-positive fibrotic areas were also markedly reduced (0.34 ± 0.21 vs. 2.5 ± 0.9%, *P* < 0.001, Fig. 1A,C). In addition, O.R.O staining-positive lipid droplet areas were reduced (Fig. 1A). The NAS was 2.3 ± 1.2, and the fibrosis stage was 0.5 ± 0.5, indicating that steatohepatitis was improved (Table 1).

**Table 1:** Liver pathology score.

Parameter	C	N	NP
NAS	0.0 ± 0.0	7.3 ± 0.8^a^	2.3 ± 1.2^b^
Steatosis	0.0 ± 0.0	2.5 ± 0.5^a^	1.2 ± 0.4^b^
Lobular inflammation	0.0 ± 0.0	2.8 ± 0.4^a^	0.5 ± 0.5^b^
Hepatocyte ballooning	0.0 ± 0.0	2.0 ± 0.0^a^	0.7 ± 0.5^b^
Fibrosis	0.2 ± 0.2	3.2 ± 0.4^a^	0.5 ± 0.5^b^

The nonalcoholic fatty liver disease activity score (NAS) and fibrosis stage were scored.

Results are presented as the mean ± SD, and p values were calculated using one-way ANOVA with Tukey’s post hoc test. ^a^*P* < 0.001, C vs. N group or ^b^*P* < 0.001, N vs. NP group. The number of mice in each group: C, n = 6; N, n = 6; NP, n = 6.

To characterize hepatic lipid contents, we conducted a lipid extraction analysis. The TC and FC levels in the liver were highly elevated in the N group compared to the C group (TC: 31.2 ± 1.2 vs. 3.1 ± 0.3 mg/g liver, *P* < 0.001, and FC: 13.1 ± 1.8 vs. 3.0 ± 0.3 mg/g liver, *P* < 0.001, Fig. 1D). On the other hand, these levels were decreased by approximately 50% in the NP group compared to the N group (TC: 13.8 ± 2.2 vs. 31.2 ± 1.2 mg/g liver, *P* < 0.001, and FC: 7.7 ± 1.4 vs. 13.1 ± 1.8 mg/g liver, *P* < 0.001, Fig. 1D). TG in the liver was not significantly lower in the NP group than in the N group, which is consistent with the findings of Araki et al. (Fig. 1D)^18^. Since cholesterol crystals are considered to be cytotoxic and cause inflammation, we determined whether cholesterol crystals might accumulate in the liver by polarizing microscopy. Marked accumulation of cholesterol crystals was observed around the central vein in the N group, while the accumulation was suppressed in the NP group (Fig. 1E).

To further investigate the events occurring in the liver, quantitative PCR and RNA sequencing of the liver were performed. The mean value of the expression of the C group, obtained by correcting each CT value with the respective housekeeping gene, was used as a control (one) to indicate the relative expression. In the N group compared to the C group, the expression of genes related to inflammation, such as *Tnfa*, *Il1b,* and *Il6*, and fibrosis, such as *Tgfb*, *Col1a1*, and *Col1a2*, were upregulated, while the expression in the NP group compared to the N group was downregulated. In the NP group compared to the N group, the expression of genes involved in liver β-oxidation, such as *Ucp3*, *Cpt1a*, and *Acox*, was upregulated. *Hmox1* and *Acat1* were downregulated by 70% in the N group compared to the C group. In addition to these genes, *Fgf21*, which increases fatty acid oxidation in the liver^18^, was upregulated by 350% in the NP group compared to the N group. Importantly, the gene expression of *Ccl5,* which has been reported to be a chemokine that induces hepatic fibrosis^22^, and *Ccl2, Caspase-1,* and *Nlrp3*, which are involved in inflammasome activation, was also upregulated in the N group compared to the C group and downregulated in the NP group compared to the N group (Fig. 1F).

The effect of pemafibrate on steatohepatitis was investigated by examining the expression of autophagy-related proteins. In the NP group compared to the N group, LC3-II/LC3-I was lower, and the protein expression levels of Rubicon, p62, and Beclin-1 were not significantly changed. (Supplemental Fig. 1A ,B, full-length blots/gels are presented in Supplemental Fig. 2). This finding suggests that autophagy was promoted by the accumulation of cholesterol but inhibited by pemafibrate.

**Figure 2:**
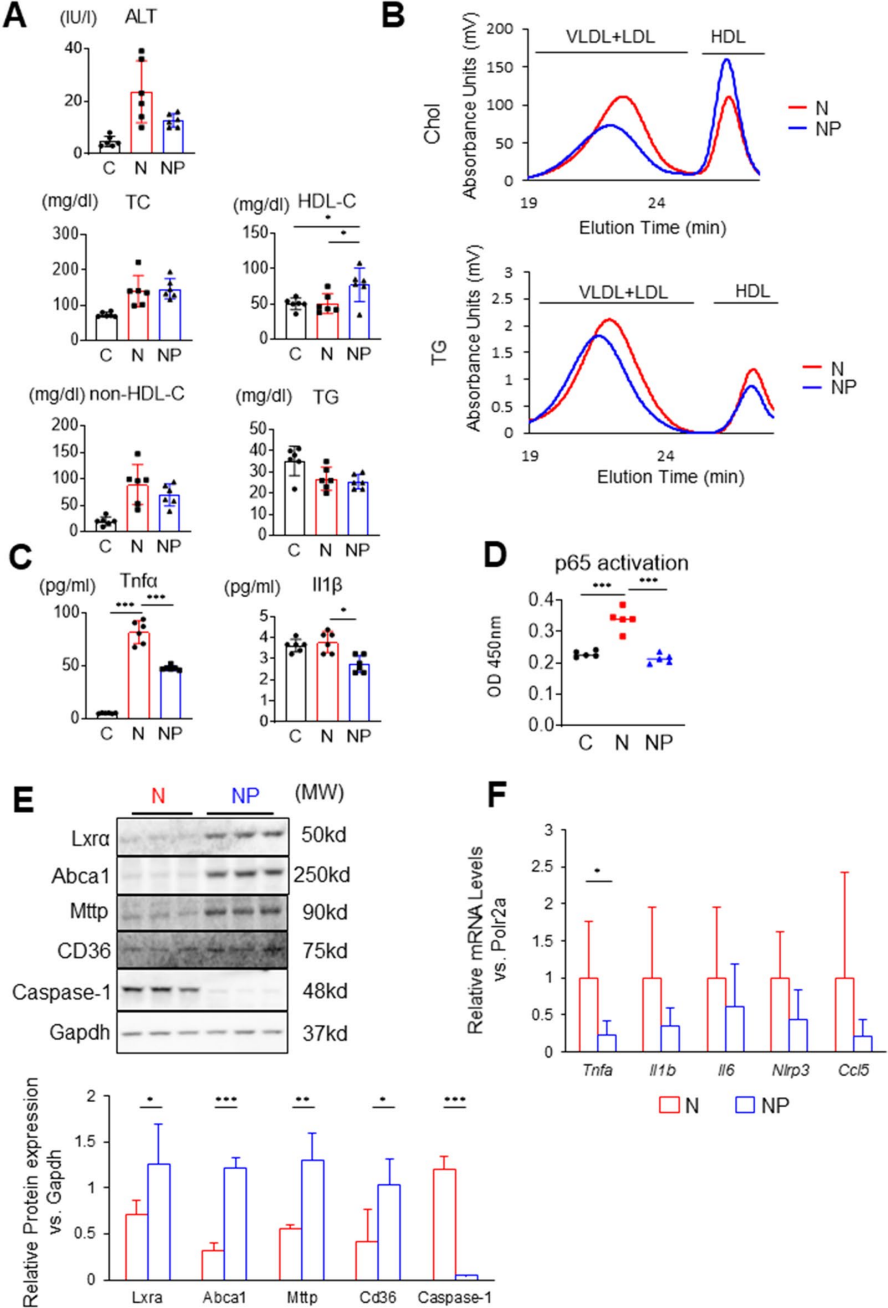
Serum lipid profiles, cytokines, hepatic protein expression, and gene expression in stellate cells. (**A**) Serum ALT and lipid profiles. (**B**) Serum high-performance liquid chromatography for cholesterol or triglycerides (six sera combined). (**C**) Serum TNF-α and IL-1β were measured by enzyme-linked immunosorbent assay. (**D**) Nucleoprotein was extracted from the liver (Nuclear Extract Kit; Active Motif), and NF-κB p65 activity was measured (TransAM NF-kB p65 Chemi; Active Motif). (**E**) Liver western blots for Lxrα, Abca1, Mttp, CD36, caspase-1, Akt, and Gapdh. Full-length blots are presented in Supplemental Fig. 3. Images were scanned and quantified by ImageJ software. (**F**) Liver stellate cells were isolated and examined for gene expression of *Tnfa*, *Il1b*, *Il6*, *Nlrp3*, and *Ccl5*. Results are presented as the mean ± SD, and p values were calculated using one-way ANOVA with Tukey’s post hoc test (**A**–**D**), Student’s t test (**E**), and Welch's t test (Fig. 1F). *P < 0.05, **P < 0.01 and ***P < 0.001, C vs. N group or N vs. NP group. The number of mice in each group: C, n = 6; N, n = 6; NP, n = 6. ALT, alanine aminotransferase; TC, total cholesterol; HDL-C, high-density lipoprotein cholesterol; TG, triglycerides; MW, molecular weight.

### Serum lipid profiles were improved, and cytokine-related gene expression was suppressed by pemafibrate

The serum HDL-C level was significantly higher in the NP group than in the C and N groups, which is consistent with a previous report by Takei et al.^23^. The serum TG levels were not significantly changed in the three groups (Fig. 2A). To analyse lipoprotein subfractions, we performed HPLC. The amounts of cholesterol in the very low-density lipoprotein (VLDL)/low-density lipoprotein (LDL)-like fractions were lower in the NP group than in the N group, and the peak cholesterol size was larger in the NP group. The amount of cholesterol in the HDL-like fraction in the NP group was 1.5 times higher than that in the N group, as calculated from the area under the curve of the HDL-C fraction of HPLC. The amounts of TG in the VLDL/LDL-like and HDL-like fractions were both lower in the NP group (Fig. 2B). The serum TNF-α increased 16-fold in the N group compared to the C group (82.0 ± 10.8 vs. 5.5 ± 0.4 pg/ml, *P* < 0.001), while it decreased by approximately 40% in the NP group compared to the N group (48.0 ± 2.1 vs. 82.0 ± 10.8 pg/ml, *P* < 0.001, Fig. 2C). The serum IL-1β was significantly decreased in the NP group compared to the N group (2.78 ± 0.44 vs. 3.68 ± 0.45, *P* < 0.05, Fig. 2C). To test the effect of pemafibrate on nuclear p65 activation, we extracted nuclear fractions and measured p65 subunits (Active Motif, Catalogue No: 40096). NF-κB p65 in the nuclei of liver cells was increased in the N group compared to the C group (0.028 ± 0.003 vs. 0.019 ± 0.001, *P* < 0.001) but significantly decreased in the NP group compared to the N group (0.018 ± 0.001 vs. 0.028 ± 0.003, *P* < 0.001, Fig. 2D). Western blot analysis showed that the expression of LXRα and Abca1 was upregulated in the NP group (Fig. 2E). This finding was consistent with the elevation of HDL-C in serum (Fig. 2A). The expression of MTTP and CD36, whose expression is regulated by PPARα, was similarly upregulated. The expression of caspase-1, which is associated with inflammasome activity, was suppressed in the NP group (Fig. 2E). Hepatic stellate cells have been reported to promote liver fibrosis through the expression of *Ccl5*^22^. We performed RT–qPCR of stellate cells isolated from the liver. The gene expression of *Tnfa* was decreased in the NP group compared to the N group (Fig. 2F). *Il1b*, *Il6*, *Nlrp3*, and *Ccl5* in the stellate cells tended to decrease in the NP group compared to the N group (Fig. 2F). Treatment with pemafibrate increased serum HDL-C and impaired TNF-α and IL-1β. Consistently, nuclear NF-κB in the liver was decreased and the protein expression of Caspase-1 was suppressed.

### Steatohepatitis-related cardiomyopathy detected by cardiac MRI was characterized by moderate myocardial damage

**Figure 3:**
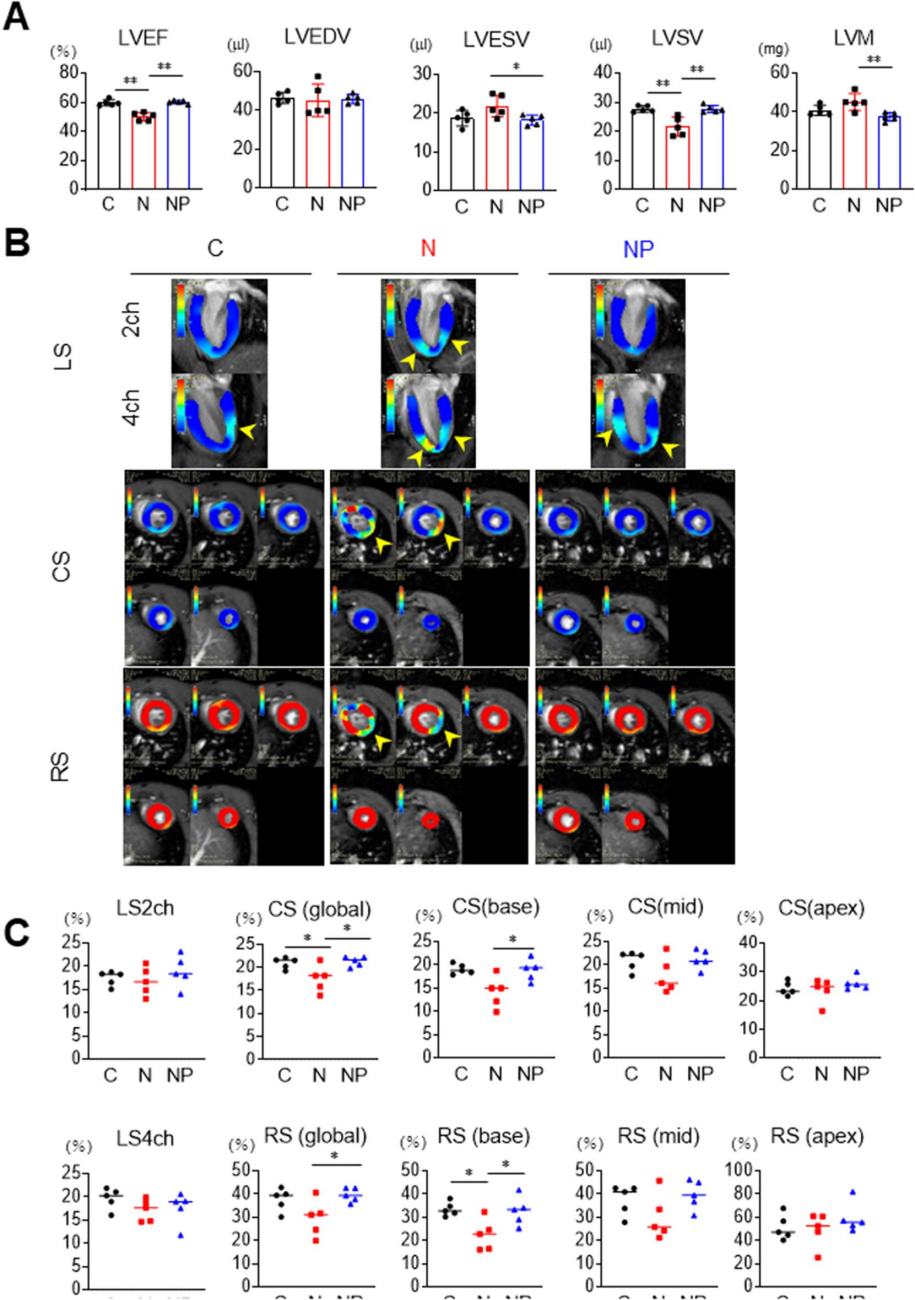
Cardiac function and LV strain were analysed by ultrahigh magnetic field 7-T MRI. (**A**) LVEF, LVEDV, LVESV, LVSV, and LVM were measured in the C, N, and NP groups. (**B**) and (**C**) myocardial strain values in three directions (longitudinal, circumferential, and radial) were analysed using cvi42 software. The yellow arrowheads indicate areas of decreased LV strain. The results are presented as the mean ± SD, and p values were calculated using one-way ANOVA with Tukey’s post hoc test. *P < 0.05, **P < 0.01, C vs. N group or N vs. NP group. The number of mice in each group: C, n = 5; N, n = 5; NP, n = 5. LVEF, left ventricle ejection fraction; LVEDV, left ventricular end-diastolic volume; LVESV, left ventricular end-systolic volume; LVSV, left ventricular stroke volume; LVM, left ventricular mass; LS, longitudinal strain; CS, circumferential strain; RS, radial strain.

Cardiac function was investigated using ultrahigh magnetic field 7-T MRI. LV wall motion disturbance, mass, and attenuation of strain were measured using a workstation. The LV ejection fraction (LVEF) was decreased in the N group compared to the C group (N; 50.2 ± 2.8 vs. C; 59.8 ± 2.1%, respectively, *P* < 0.001, Fig. 3A). On the other hand, it was significantly higher in the NP group than in the N group (NP; 61.4 ± 1.1 vs. N; 50.2 ± 2.8%, respectively, *P* < 0.001, Fig. 3A). Left ventricular end-diastolic volume (LVEDV) did not differ among the three groups (C; 46.5 ± 2.8 vs. N; 45.2 ± 8.3 vs. NP; 48.4 ± 2.3 µl, respectively, Fig. 3A). In the N group, left ventricular end-systolic volume (LVESV) was increased compared to the C group (N; 22.4 ± 3.6 vs. C; 18.8 ± 2.0 µl, respectively, *P* = 0.07, Fig. 3A), resulting in a significant decrease in left ventricular stroke volume (LVSV) compared to the C group, calculated as LVEDV-LVESV (N; 22.8 ± 5.0 vs. C; 27.7 ± 1.1 µl, respectively, *P* < 0.01, Fig. 3A). In the NP group, LVESV was lower and LVSV was increased compared to the N group (LVESV: NP; 19.6 ± 1.3 vs. N; 22.4 ± 3.6 µl, respectively, *P* < 0.05 and LVSV: NP; 28.8 ± 1.2 vs. N; 22.8 ± 5.0 µl, respectively, *P* < 0.01, Fig. 3A). LV mass (LVM) tended to increase in the N group compared to the C group (N; 45.2 ± 4.4 vs. C; 40.1 ± 2.7 mg, respectively, *P* = 0.1). On the other hand, it was significantly lower in the NP group than in the N group (NP; 39.6 ± 2.1 vs. N; 45.2 ± 4.4 mg, respectively, *P* < 0.01, Fig. 3A).

Correctively, between the C group and the N group, we observed a moderate reduction of LVEF and LVSV, no difference in LVEDV, and an increasing trend of LVM, suggesting that steatohepatitis-related cardiomyopathy was characterized by modestly disturbed LV wall motion with concentric hypertrophy. In addition, these phenotypes were completely suppressed by pemafibrate (Fig. 3A).

Cardiac strain describes the deformation of the cardiac wall or chamber from a relaxed to a contracted condition. To further characterize steatohepatitis-related cardiomyopathy, we measured longitudinal strain (LS), circumferential strain (CS), and radial strain (RS). The blue colour in LS and CS and the red colour in RS represent normal cardiac strain. Interestingly, representative images revealed that there were focal low-strain areas in the left ventricle (indicated by the yellow arrows in Fig. 3B), suggesting the development of cardiomyopathy under the background of steatohepatitis. In LS, there were no differences in LS2ch and LS4ch between the 3 groups (Fig. 3C). The percentage of global CS in the N group was attenuated compared to that in the C group (N; 17.5 ± 2.9 vs. C; 20.9 ± 1.2%, *P* < 0.05), and that in the NP group was higher than that in the N group (NP; 20.2 ± 1.1 vs. N; 17.5 ± 2.9%, *P* < 0.05, Fig. 3C). These changes in CS (base) were more obvious. The percentage of CS (base) in the NP group was higher than that in the N group (NP; 16.6 ± 2.3 vs. N; 14.2 ± 3.4%, *P* < 0.05, Fig. 3C). Similarly, the percentage of RS (global) in the NP group was higher than that in the N group (NP; 42.7 ± 3.0 vs. N; 29.7 ± 7.8%, *P* < 0.05, Fig. 3C). The percentage of RS (base) in the N group was attenuated compared to that in the C group (N; 22.5 ± 6.7 vs. C; 33.6 ± 3.0%, *P* < 0.05), and that in the NP group was higher than that in the N group (NP; 38.9 ± 6.2 vs. N; 22.5 ± 6.7%, *P* < 0.05, Fig. 3C). On the other hand, there was no significant difference in CS (apex) and RS (apex) among the three groups.

### Steatohepatitis-related cardiomyopathy was associated with cardiac hypertrophy, free cholesterol accumulation, and inflammatory cell infiltration

**Figure 4:**
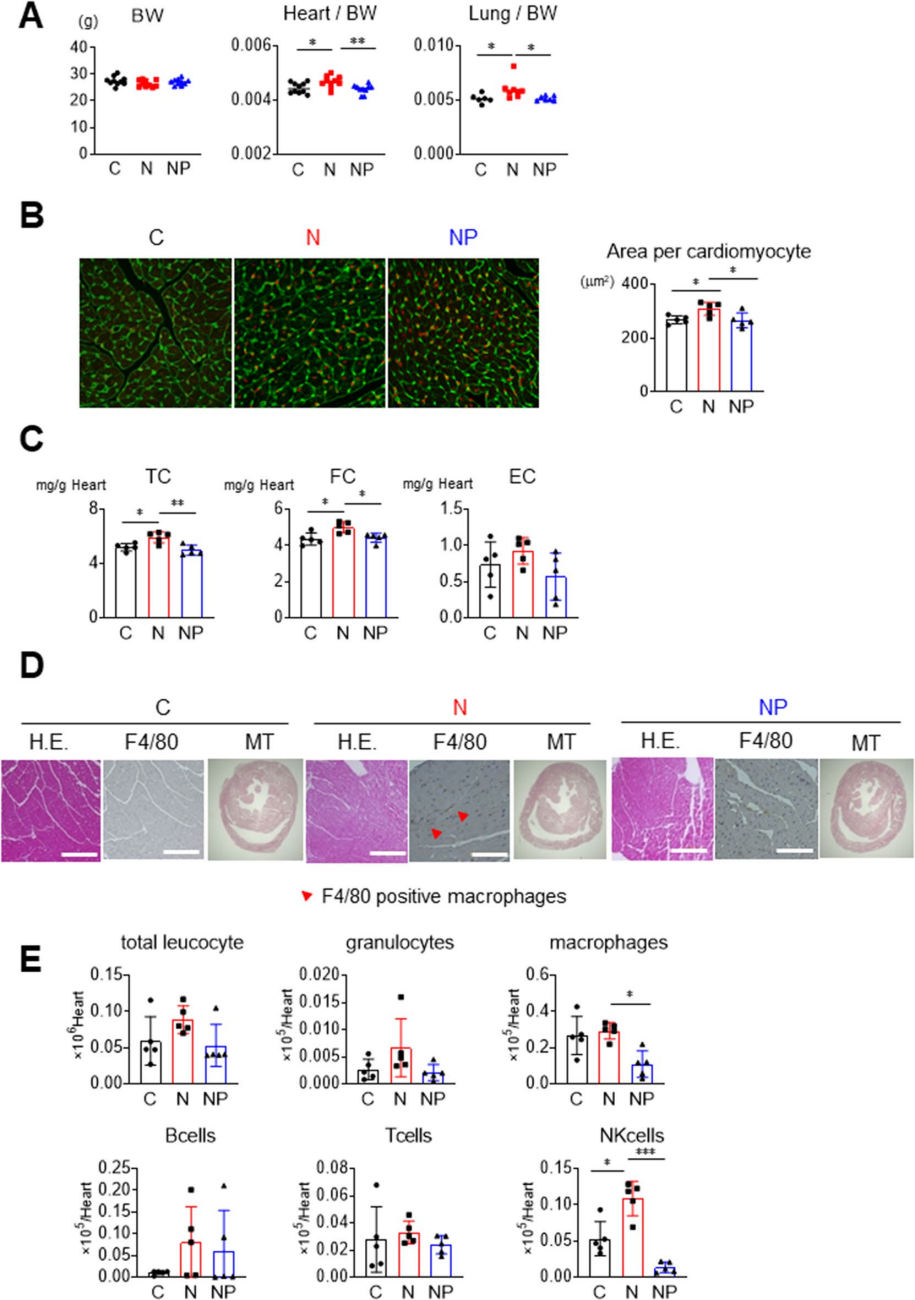
Cardiac phenotypes and histological images. (**A**) Body weight, heart/body weight, and lung/body weight were measured. (**B**) Cardiac WGA staining was performed, and the mean area per cardiomyocyte was measured. (**C**) Cardiac cholesterol contents were measured. (**D**) Cardiac H.E., F4/80, and MT staining in each group. The red arrows indicate the infiltration sites of F4/80-positive macrophages. (**E**) Flow cytometry analysis of cells isolated from collagenase-treated hearts. Myocardial leukocyte fraction was measured. The results are presented as the mean ± SD, and p values were calculated using one-way ANOVA with Tukey’s post hoc test. *P < 0.05, **P < 0.01 and ***P < 0.001; C vs. N group or N vs. NP group. The number of mice in each group: C, n = 10 (A), n = 5 (B-E); N, n = 10 (A), n = 5 (B-E); NP, n = 10 (A), n = 5 (B-E). WGA, wheat germ agglutinin; EC, esterified cholesterol; H.E., haematoxylin–eosin; M.T., Masson's trichrome.

Steatohepatitis-related cardiomyopathy was identified by examining tissue weight, mean cardiomyocyte area, lipid content, and histology. There were no differences in body weight among the 3 groups (Fig. 4A). Compared to the C group, the heart weight to body weight ratio and lung weight to body weight ratio were higher in the N group but similar to those in the NP group (Fig. 4A). Wheat germ agglutinin staining showed that the mean cardiomyocyte area was higher in the N group than in the C group (308 ± 25 vs. 267 ± 15 µm^2^, respectively, *P* < 0.05), whereas there was no difference between the NP group and the C group (265 ± 28 vs. 267 ± 15 µm^2^, respectively, *P* < 0.05, Fig. 4B), suggesting hypertrophic modification of cardiomyocytes in the N group. Regarding myocardial lipid content, the heart concentrations of TC and FC were significantly higher in the N group than in the C group (TC: 5.9 ± 0.4 vs. 5.2 ± 0.3 mg/g heart, respectively, *P* < 0.05; and FC: 5.0 ± 0.3 vs. 4.5 ± 0.4 mg/g heart, respectively, *P* < 0.05, Fig. 4C). Importantly, the myocardial concentrations of TC and FC in the NP group were significantly lower than those in the N group (TC: 5.0 ± 0.4 vs. 5.9 ± 0.4 mg/g heart, respectively, *P* < 0.01; and FC: 4.4 ± 0.3 vs. 5.0 ± 0.3 mg/g heart, respectively, *P* < 0.05, Fig. 4C). Infiltration of F4/80-positive macrophages was increased in the N group compared to the C group (Fig. 4D), which was confirmed by flow cytometry analysis of cells isolated from collagenase-treated hearts (Fig. 4E). Infiltration of macrophages was suppressed in the NP group compared to the N group (0.11 ± 0.08 vs. 0.29 ± 0.05 × 10^5^/heart, *P* < 0.01, Fig. 4E). Natural killer cells were also attenuated in the NP group compared to the N groups (0.015 ± 0.008 vs. 0.11 ± 0.03 × 10^5^/heart, *P* < 0.001, Fig. 4E). Masson trichrome (MT) staining revealed no obvious fibrosis in any of the 3 groups (Fig. 4D).

### Gene and protein expression levels related to myocardial inflammasomes and cardiac hypertrophy were increased in the N group

**Figure 5:**
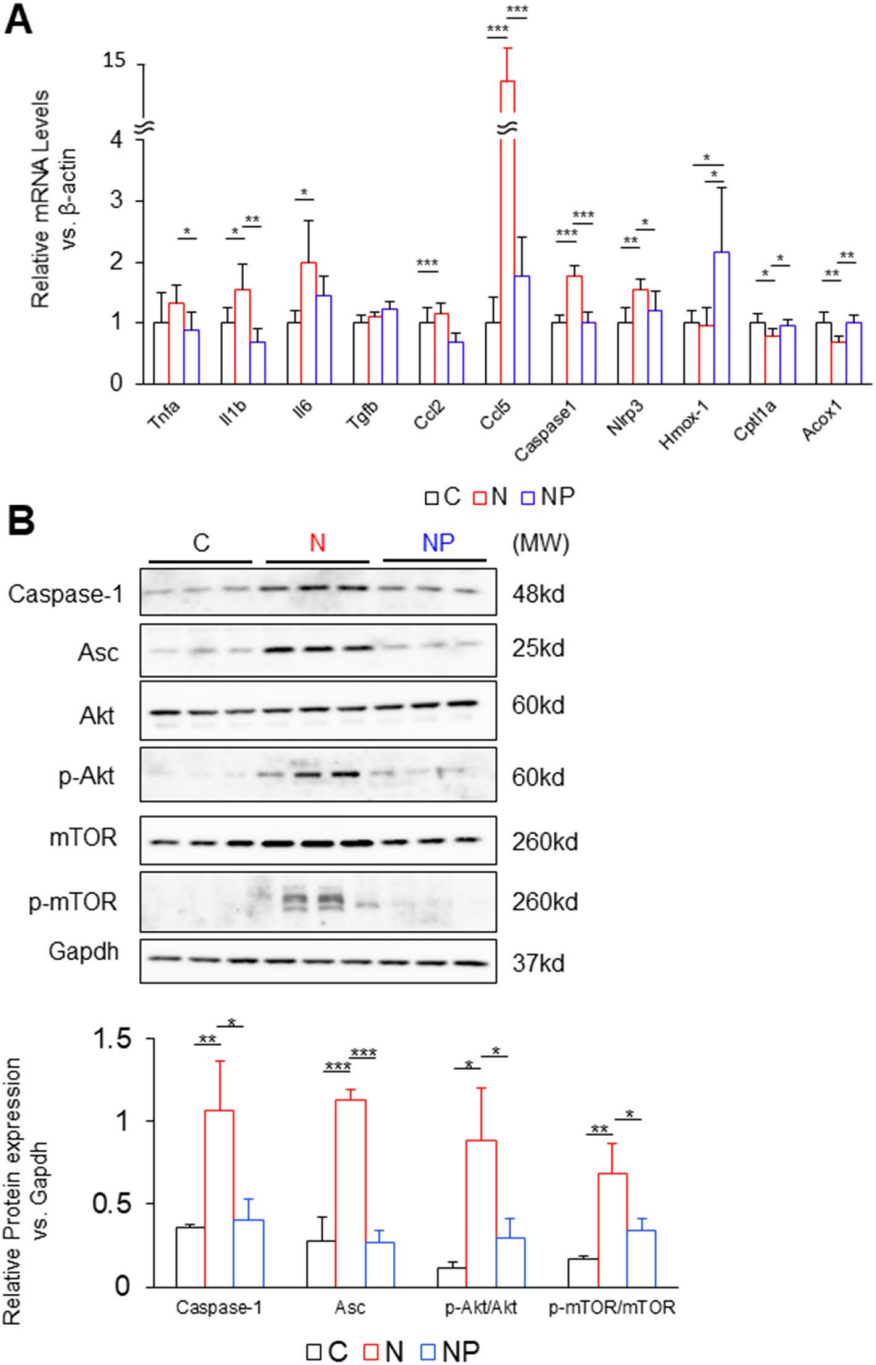
Gene expression and protein expression in the heart. (**A**) mRNA expression levels related to inflammation, fibrosis, chemokines, inflammasomes, and β-oxidation were assessed. The mean value of the expression of the C group, obtained by correcting each CT value with the respective housekeeping gene, was used as a control (one) to indicate the relative expression. (**B**) Cardiac western blots for caspase-1, Asc, Akt, p-Akt, mTOR, p-mTOR and Gapdh. Full-length blots are presented in Supplemental Fig. 4. Images were scanned and quantified by ImageJ software. The results are presented as the mean ± SD, and *P* values were calculated using one-way ANOVA with Tukey’s post hoc test. *P < 0.05, **P   < 0.01 and ***P < 0.001, C vs. N group or N vs. NP group. The number of mice in each group: C, n = 5; N, n = 5; NP, n = 5. MW, molecular weight.

In the N group compared to the C group, quantitative PCR analysis in the heart revealed that the gene expression of inflammatory cytokines and chemokines, such as *Il1b*, *Il6*, and *Ccl5*, was upregulated. *Caspase-1* and *Nlrp3* were also upregulated. In the NP group compared to the N group, *Tnfa*, *Il1b*, *Ccl2*, *Ccl5*, *Caspase-1*, and *Nlrp3* were suppressed. *Hmox-1* was upregulated in the NP group compared to the C and N groups. The expression levels of *Cpt1a* and *Acox1* were downregulated in the N group compared to the C group and upregulated in the NP group compared to the N group (Fig. 5A).

Western blot analysis revealed that the protein expression of caspase-1 and apoptosis-associated speck-like protein containing a CARD (Asc), which is a component protein of the inflammasome, was upregulated in the N group compared to the C group and downregulated in the NP group compared to the N group (Fig. 5B), suggesting an association with the inflammasome activation pathway. Since previous studies demonstrated that inflammasome activation was associated with cardiac remodelling^24,25^, the activation of Akt and mTOR was also examined. As a result, p-Akt/Akt and p-mTOR/m-TOR were increased in the N group compared to the C group (p-Akt/Akt; *P* < 0.05 and p-mTOR/mTOR; *P* < 0.01, Fig. 5B) and reduced in the NP group compared to N group (p-Akt/Akt; *P* < 0.05 and p-mTOR/mTOR; *P* < 0.05, Fig. 5B). These data suggest that free cholesterol accumulation in the heart activates inflammasomes, resulting in upregulation of the PI3K-Akt pathway. Reactive oxygen species (ROS) production in the myocardium was also confirmed by fluorescence using dihydroethidium. Compared to the C group, myocardial ROS were detected more strongly in the N group and less strongly in the NP group (Supplemental Fig. 5A).

### Pathways related to NF-κB signalling, the NOD-like receptor, and PI3K-Akt were upregulated in steatohepatitis and suppressed by pemafibrate

To understand the molecular mechanisms in the heart, we performed RNA sequencing. The pathway analysis demonstrated that pathways involved in inflammation, such as cytokine–cytokine receptor interaction, chemokine signalling pathway, NOD-like receptor signalling pathway, and PI3K-Akt signalling pathway, tended to be upregulated in the N group compared to the C group (Supplemental Fig. 6A). In the heatmap of the C, N, and NP groups, upregulated expression was observed for genes involved in the NF-κB signalling pathway, the NOD-like receptor signalling pathway, which is associated with inflammasome activation, and the PI3K-Akt signalling pathway, which is related to cardiac hypertrophy, in the N group compared to the C group, while downregulated expression was observed in the NP group (Supplemental Fig. 6B).

**Figure 6:**
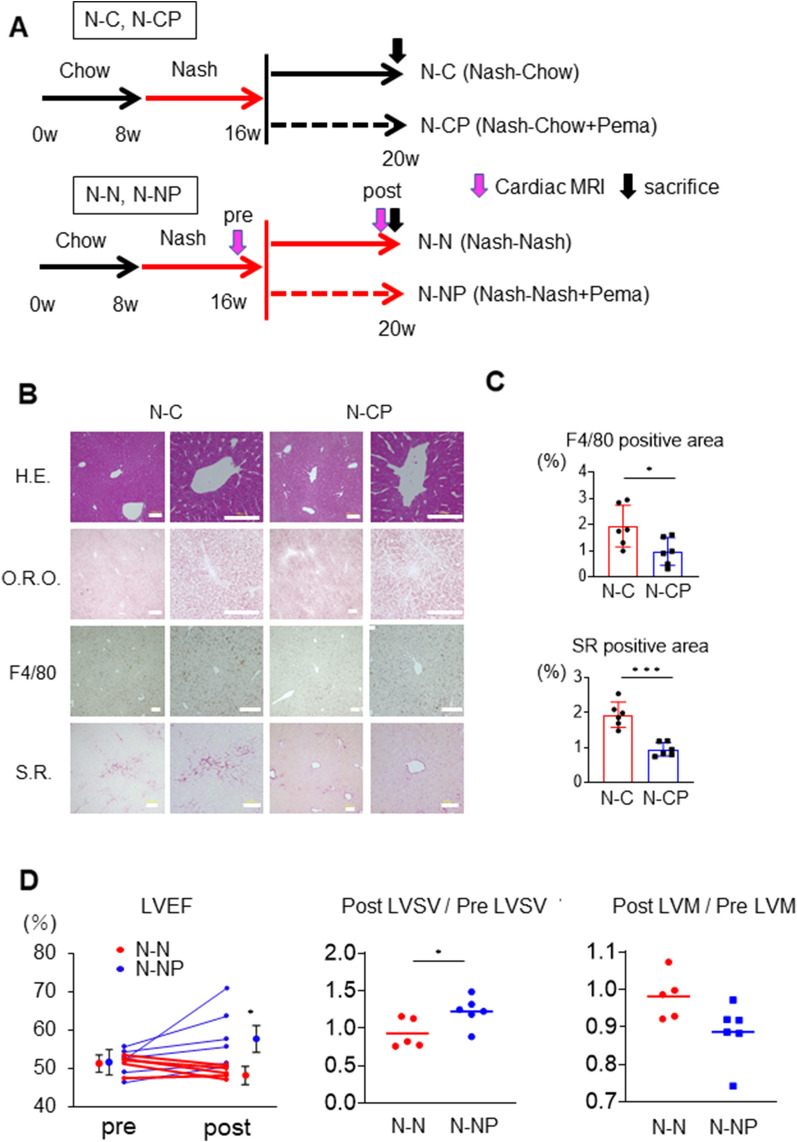
Effect of pemafibrate after the development of steatohepatitis and cardiomyopathy. (**A**) Chow diet, chow diet + 0.1 mg/kg pemafibrate, NASH diet, or NASH diet + 0.1 mg/kg pemafibrate diet was fed to C57Bl6 male mice for 4 weeks after feeding the NASH diet for 8 weeks (N–C, N-CP, N–N, and N-NP groups, respectively). Cardiac MRI was performed before and after the additional diet in the N–N and N-NP groups. (**B**) Liver H. E, O.R.O., F4/80, and S.R. staining in N–C and N-CP groups. (**C**) Percentages of examined areas positive for F4/80 and Sirius Red. (**D**) LVEF, LVSV, and LVM in N–N and N-NP groups. Pre- and post-LVEF, the ratios of post/pre LVSV and post/pre LVM are shown. Scale bar: 100 µm. The results are presented as the mean ± SD, and p values were calculated using Student’s t test. *P < 0.05, N–C vs. N-CP or N–N vs. N-NP group. The number of mice in each group: N–C, n = 6; N-CP, n = 6; N–N, n = 6; N-NP, n = 6. H.E., haematoxylin–eosin; O.R.O., Oil Red O; S.R., Sirius Red; LVEF, left ventricle ejection fraction; LVSV, left ventricular stroke volume; LVM, left ventricular mass.

### Pemafibrate reversed steatohepatitis and cardiomyopathy

To test whether pemafibrate could regress pre-existing steatohepatitis, the N diet was fed to mice for 8 weeks, followed by the C diet or C diet + pemafibrate (CP diet) for 4 weeks (N–C and N-CP groups, respectively, Fig. 6A). Histological examination of the N–C and N-CP groups showed that macrophage infiltration was reduced in the N-CP group (1.93 ± 0.79 vs. 0.97 ± 0.53%, *P* < 0.05, Fig. 6B,C). There was no difference in the area of lipid droplets between the two groups (Fig. 6B). Importantly, the SR staining-positive fibrosis area was significantly smaller in the N-CP group than in the N–C group (0.95 ± 0.20 vs. 1.94 ± 0.36% *P* < 0.05, Fig. 6B,C). Similarly, to test whether pemafibrate could ameliorate pre-existing cardiac dysfunction, mice received the N diet for 8 weeks and subsequently the N diet or NP diet for 4 weeks (N–N and N-NP groups, respectively, Fig. 6A). Cardiac LVEF, LVSV, and LVM were measured by MRI before and after administration of the N–N or N-NP diet (pre, post, respectively). Notably, LVEF was improved by the administration of pemafibrate in the N-NP group compared to the N–N group (post-LVEF: 58 ± 8 vs. 49 ± 2%, *P* < 0.05, Fig. 6D). In addition, the post-LVSV/pre-LVSV values were higher in the N-NP group than in the N–N group (1.23 ± 0.20 vs. 0.93 ± 0.20, *P* < 0.05, Fig. 6D), and the post-LVM/pre-LVM values were numerically lower in the N-NP group than in the N–N group (0.87 ± 0.08 vs. 0.98 ± 0.06, *P* = 0.055, Fig. 6D). Pemafibrate ameliorated hepatic fibrosis and cardiac dysfunction even after steatohepatitis and steatohepatitis-related cardiomyopathy had developed.

## Discussion

**Figure 7:**
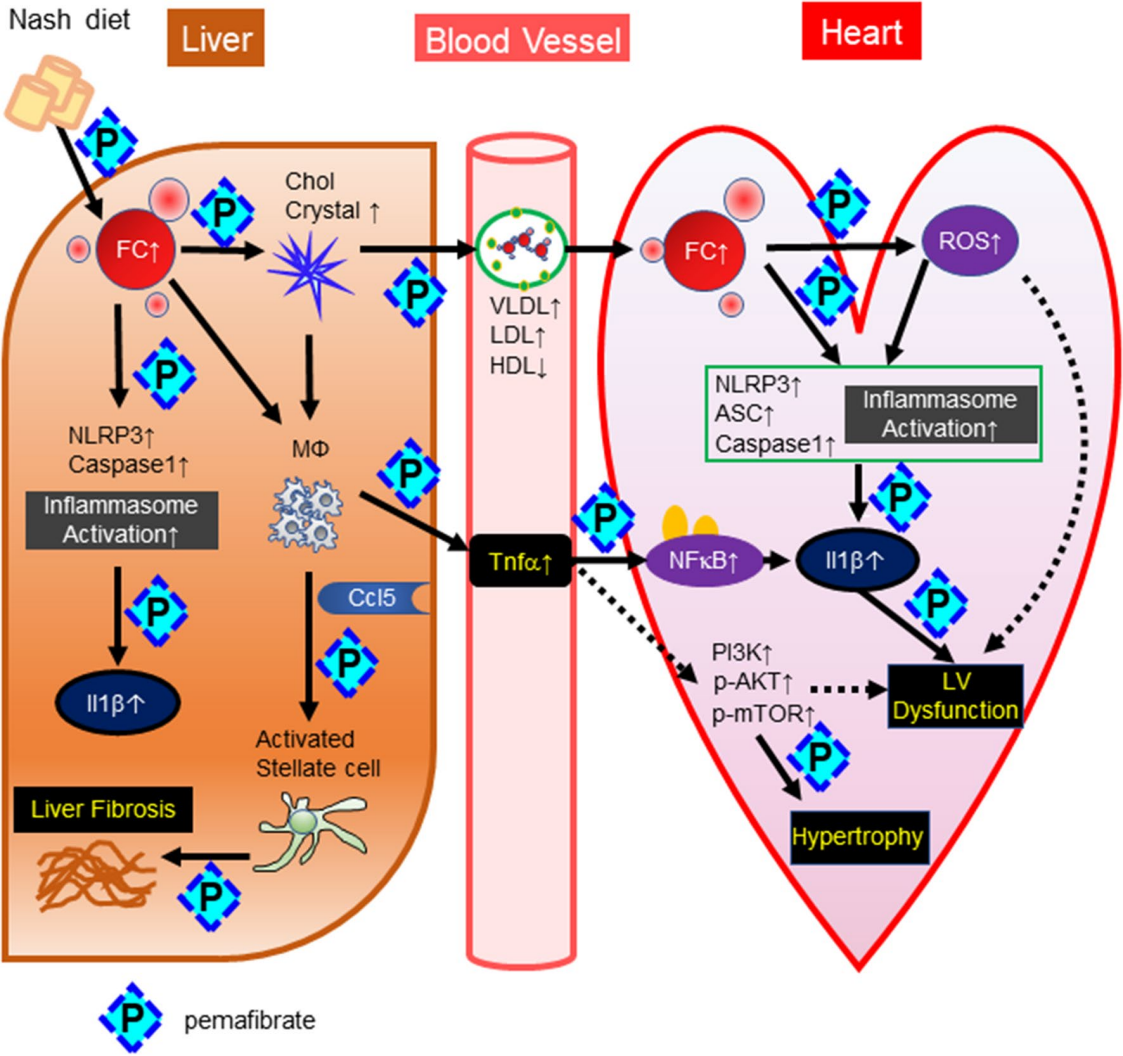
Mechanisms of steatohepatitis-related cardiomyopathy and the effects of pemafibrate. Cholesterol absorbed from the NASH diet is first taken up by the liver. Some of the free cholesterol in the liver becomes cholesterol crystals, which are taken up by macrophages and hepatocytes and lead to the production of IL-1β. In addition, macrophages produce Ccl5, which activates stellate cells and causes liver fibrosis. Free cholesterol in VLDL and LDL is taken up into the myocardium, where it activates macrophages and inflammasomes of myocardial cells, induces IL-1β production, and causes LV hypertrophy and dysfunction. Furthermore, ROS generated by an increase in free cholesterol are also considered to be involved in cardiac dysfunction. Pemafibrate reduces the level of free cholesterol in the liver and inhibits the production of cholesterol crystals, thereby suppressing the activity of inflammasomes and inhibiting the development of steatohepatitis. As a result, serum VLDL, LDL, and TNF-α were reduced and HDL was increased. Free cholesterol in the myocardium was reduced and inhibited myocardial damage via suppression of myocardial inflammasome activity.

Recent clinical studies have demonstrated that NAFLD is closely associated with cardiac dysfunction or atrial fibrillation^7,8,26^. Larger rodent animals, such as hamsters and rats, with steatohepatitis accompanied by cardiomyopathy have been reported^27,28^ ; however, there is no report regarding a mouse model. In the current study, we developed a high fat/high cholesterol/high sucrose/bile acid diet^29^. In this model, steatohepatitis was characterized by more accumulation of free cholesterol and cholesterol crystals than other steatosis models, such as *ob/ob* mice fed a high-fat diet^30^, thus leading to activation of the inflammasome pathway, including NLRP3 and IL-1β (Fig. 7). In the heart, we also found free cholesterol accumulation (Fig. 4C), which might be involved in the upregulation of Caspase-1, Asc, and IL-1β and the activation of the NLRP3 inflammasome pathway (Fig. 7). Takahashi et al. previously reported that NLRP3 and inflammasome activation were involved in myocardial remodelling after myocardial infarction^31^. Xiao et al. also demonstrated that inflammasome-dependent activation of IL-18 was observed within the myocardium in an isoproterenol-induced cardiomyopathy model, suggesting the involvement of inflammasome activation in cardiomyocytes^32^. We also showed LV concentric hypertrophy and a reduction in LVEF and LV strain. RNA sequence analysis and western blot analysis demonstrated that the PI3K-Akt pathway was upregulated (Fig. 5B). These results were consistent with previous reports demonstrating that activation of PI3K, Akt, and mTOR was involved in cardiac hypertrophy and dysfunction^24,25^. There are several possible mechanisms linking steatohepatitis and cardiac inflammation, including the following: (1) abnormal lipid profiles in serum, such as an increase in Apo B-containing lipoproteins and a decrease in the HDL fraction; (2) enhancement of inflammatory cytokines and chemokines in serum, such as TNF-α, IL-1β, and CCL5; and (3) hepatokines, such as fetuin-A, retinol-binding protein 4, and selenoprotein P^33–35^. In our mouse model of the current study, there is a trend of increase in TC and non-HDL-C, which are Apo B-containing lipoproteins in the N group compared to the C group. Also, there is no change in HDL-C and TG in the N group compared to the C group. Those are not fully consistent with the common lipid profiles in the human subjects with NAFLD. This might be a limitation of this model. However, we are considering that some particular patients with NASH might share these mechanisms. Further clinical studies investigating patients with NASH and cardiac dysfunction will be required.

Interestingly, *Ccl5,* a member of the CC chemokine family, was highly expressed in both the liver and heart in the N group. Liver fibrosis has been reported to be exacerbated by Ccl5 secreted by HSCs^22^. Teratani et al. showed that the accumulation of free cholesterol in HSCs resulted in the activation of these cells and induced fibrosis-related genes^36^. Ccl5 has also been reported to be involved in postmyocardial infarction remodelling^37^. Therefore, we are still deliberating that Ccl5 might contribute to the progression of LV diastolic dysfunction by acting on cardiac fibroblasts as it does on the liver. Further investigation is required. We also speculated a couple of putative mechanisms between steatohepatitis and cardiac hypertrophy. Since epidermal growth factor (EGF), vascular endothelial growth factor (V-EGF) and receptor tyrosine kinase have been reported to stimulate the PI3K-AKT pathway and induce LV hypertrophy^38^, we speculate that some hepatokines secreted by steatohepatitic cells might stimulate one of these pathways.

The characteristics of steatohepatitis-associated cardiomyopathy in this study were concentric LV hypertrophy, modest reduction in LVEF, and regional reduction in myocardial strain, all of which could be detected by cardiac MRI and strain analysis. In the present study, we detected focal attenuation of LV strain, although whether this reflects simple dysfunction of cardiomyocytes or perhaps myocardial disarray, such as that seen in hypertrophic cardiomyopathy, has not been clarified^39^. Although these phenotypes were observed after only eight weeks of administration of the N diet, a longer study might be able to reveal more severe phenotypes, such as heart failure with preserved ejection fraction. Since novel methods for detecting cardiac fibrosis, such as T1 mapping and extracellular volume fraction, have been recently developed, it might be beneficial to use cardiac MRI to diagnose individuals with steatohepatitis-related cardiomyopathy. We believe that cardiac MRI may have an advantage in detecting metabolic cardiomyopathies such as steatohepatitis-related cardiomyopathy and diabetic cardiomyopathy^40^.

Pemafibrate has been developed against hypertriglyceridemia. In addition, Nakajima et al. recently reported that pemafibrate did not decrease liver fat content but had a significant reduction in magnetic resonance elastography-based liver stiffness^41^. Pemafibrate has also been reported to have various effects on systemic organs in animal studies. Pemafibrate suppressed body weight gain in a mouse model of diet-induced obesity^18^. This effect was explained by upregulation of the genes involved in thermogenesis and fatty acid oxidation, such as *Ucp1*, *Cidea*, and *Cpt1b* in inguinal adipose tissue and *Elovl3* in brown adipose tissue^18^. Moreover, Honda et al. demonstrated that pemafibrate attenuated hepatic TG content, macrophage infiltration, and fibrosis in an amylin liver NASH mouse model^19^. These findings were explained by β-oxidation caused by marked upregulation of *Ucp3* in the liver^19^. Our previous study also demonstrated that intestinal lipid absorption was suppressed by pemafibrate^42^. In this study, our steatohepatitis model demonstrated a rapid progression of hepatic fibrosis, even at 8 weeks, and pemafibrate completely suppressed *Col1a1*, *Col1a2*, and *Ccl5* mRNA, which are involved in fibrosis. Since anti-hepatic fibrosis drugs have not yet been developed, pemafibrate might be considered an alternative. Regarding the impact of fibrates on the heart, one study found that fenofibrate exerted a protective myocardial effect in autoimmune myocarditis rats^21^, and the authors speculated that fenofibrate induced an increase in IL-10^21^. A previous report using COS cells revealed that PPARα agonists inhibited NF-κB transactivation, which might contribute to suppression of the vascular inflammatory response^43^. In the present study, we first demonstrated that pemafibrate suppressed cholesterol accumulation in the heart and inhibited NLRP3 inflammasome activation, IL-1β expression, and the PI3K-Akt pathway, resulting in preserved LV motion and LV strain (Fig. 3). Importantly, sequential administration of pemafibrate after an 8-week N diet resulted in suppression of hepatic inflammation and fibrosis as well as amelioration of LVEF, indicating that pemafibrate has a beneficial effect on pre-existing steatohepatitis-related cardiomyopathy (Fig. 6). We expect that pemafibrate might alleviate hypertriglyceridemia, NASH, and NASH-related cardiomyopathy. Further randomized clinical trials using pemafibrate and cardiac MRI will be required to prove this hypothesis.

## Conclusions

We report the first model of steatohepatitis-related cardiomyopathy characterized by activation of the NLRP3 inflammasome pathway. Pemafibrate prevented and ameliorated both hepatic fibrosis and cardiac dysfunction by inhibiting inflammasome pathway activation.

## Methods

### Animals and diets

C57BL/6 J male mice were obtained from Charles River Laboratories (Tokyo, Japan), housed in a temperature- and humidity-controlled facility with a 12-h light/dark cycle, and fed 3 types of diet for 8 weeks. A normal chow diet (C diet) was fed to the C group, a diet containing bile acid and high concentrations of fat, cholesterol, and sucrose (casein 20%, sucrose 50%, cocoa butter 15%, cholesterol 1.25%, cholate 0.5%; N diet) was fed to the N group, and the N diet with 0.1 mg/kg pemafibrate (N diet + pemafibrate; NP diet) was fed to the NP group. When sacrificing mice, they were anaesthetized by an intraperitoneal injection of medetomidine (0.3 mg/kg), midazolam (4 mg/kg), and butorphanol (5 mg/kg). Adequate anaesthesia was maintained by monitoring the respiration rate and the lack of response to paw pinching. After the study, all anaesthetized animals were euthanized by cervical dislocation. The experimental protocol was approved by the Ethics Review Committee for Animal Experimentation of Osaka University School of Medicine. All experiments were conducted in accordance with the Use of Laboratory Animals, which was incorporated into the Institute for Laboratory Animal Research Guide for the Care and Use of Laboratory Animals.

### Biochemical analyses

The alanine aminotransferase (ALT), total cholesterol (TC), HDL-C, and triglycerides (TG) contents in serum were measured by enzymatic methods (Fujifilm, Tokyo, Japan). Non-HDL-C was calculated as TC minus HDL-C. Lipoprotein profiles of plasma after 4 h of fasting were compared using high-performance liquid chromatography (HPLC) (Liposearch, Skylight Biotech, Tokyo, Japan). Hepatic TC, free cholesterol (FC), TG, and nonesterified fatty acids were also measured (Wako Pure Chemical Industries, Tokyo, Japan) after extraction of lipids from liver tissue by the Folch method. Esterified cholesterol was calculated as TC minus FC. Serum tumour necrosis factor-α (TNF-α) and interleukin-1β (IL-1β) levels were determined using mouse TNF-α and IL-1β ELISA kits (MTA00B and MLB00C, Quantikine, MN, USA), respectively.

### Histologic and immunohistochemical analyses

Paraffin-embedded sections were stained with haematoxylin and eosin (200,108, Muto Pure Chemicals, Tokyo, Japan) or Sirius Red (MKCB3138 V, Sigma–Aldrich, Tokyo, Japan). For lipid staining, frozen sections were stained with Oil Red O (M3G0644, Nacalai Tesque, Kyoto, Japan). Macrophages were detected by F4/80 (MCA497R, Bio–Rad, Tokyo, Japan) and Vectastain secondary antibodies (Vector Laboratories, Burlingame, CA, USA). To quantify the area of staining by Oil Red O and Sirius Red, images of 5 random fields from each section were processed using ImageJ software (National Institute of Mental Health, Bethesda, MD, USA). Each value was expressed as the percentage of the total area of each section that stained positively. The number of F4/80-positive cells was counted and averaged for 5 random fields in each section. The NAS and fibrosis stages were scored by Y.K. in a blinded manner according to the method of Kleiner et al.^44^.

### High-performance liquid chromatography

Lipoproteins in fresh-frozen serum (4 µl) were separated with tandemly connected Skylight PakLP1-AA gel permeation columns (Skylight Biotech Inc., Akita, Japan, 300 mm × 4.6 mm I.D.). The column effluent was then equally split into two lines by a microsplitter, and each effluent was allowed to react at 37 °C with the Cho and TG reagents. Absorbance at 550 nm was continuously monitored after each enzymatic reaction in two reactor coils (PTFE; 25 m × 0.18 mm I.D.). The amount of cholesterol in the VLDL + LDL- or HDL-like fractions was calculated from the area under the curve by adding cholesterol or TG to each fraction.

### Quantitative polymerase chain reaction (RT–qPCR) and western blotting

RT–qPCR and western blotting were performed as described previously^30^. Briefly, total RNA was isolated from liver tissues using the RNeasy® Mini Kit (QIAGEN, Hilden, Germany). RNA was reverse-transcribed using a SuperScript VILO cDNA Synthesis Kit (Thermo Fisher Scientific, CA, USA). RT–qPCR was performed using TaqMan Master Mix (Thermo Fisher Scientific, CA, USA) and a 7900 Sequence Detection System (Applied Biosystems, CA, USA). The specific primers used are listed in Supplemental Table 1. The antibodies used for the immunoblot are listed in Supplemental Table 2. Membranes were imaged with an Image Quant LAS 4000 camera system (GE Healthcare, IL, USA). The band intensity was quantified by ImageJ software.

### Strategy of tissue digestion and low cytometry

To generate single-cell suspensions for flow cytometry, whole hearts were freshly isolated. Heart ventricles were manually minced using scissors. Samples were enzymatically digested with 25 mg/mL liberase (MNP-S GMP Grade, Roche) and DNase I (Thermo Scientific) in RPMI and incubated on a shaker for 60 min at 37 °C. The cell suspension in PBS was refiltered through a 70 µm nylon mesh to remove connective tissue. All the samples were resuspended in FACS buffer (PBS containing 1% BSA, 0.1% sodium azide and 1 mM EDTA). Cell suspensions were stained with an established flow cytometry panel. Surface staining was performed by using commercially acquired antibody clones (conjugated with different fluorophores) as described in Supplemental Table 3. Flow cytometric analysis was performed with a FACS Canto II flow cytometer (BD Biosciences) and analysed using FlowJo 10.8 (Treestar, Ashland, OR) software (www.flowjo.com).

### Extraction of nuclear extracts and measurement of activated NF-κB p65

To extract the nuclear fraction, one hundred milligrams of frozen tissue was crushed using a mortar and pestle, with the base submerged in liquid nitrogen; the pestle was also cooled with liquid nitrogen. On ice, 3 ml ice-cold 1X hypotonic buffer containing phosphatase and protease inhibitors was added per gram of tissue, and the mixture was homogenized using a Dounce homogenizer with a large-clearance pestle for approximately 20 strokes to disrupt the tissue. Microscopic observation was performed to ensure that the cells were dissociated, and homogenization was continued. The mixture was incubated on ice for 15 min and then centrifuged for 10 min at 850 g at 4 °C. The supernatant was transferred into a prechilled microcentrifuge tube (Active Motif, CA, USA, cat# 40,010). Active NF-κB was measured using a Trans AM NF-κB p65 Transcription Factor Assay Kit (Active Motif, CA, USA, cat# 40,096) according to the manufacturer's instructions.

### RNA sequence analysis

RNA sequence analysis was conducted as described previously^30^. Briefly, sequencing was performed on an Illumina HiSeq 2500 platform in 75-base single-end mode. Illumina Casava 1.8.2 software (Illumina Inc, CA, USA) was used for base calling. The raw reads were mapped to the mouse reference genome sequences (mm10) using TopHat ver. 2.0.13^45^ in combination with Bowtie2 ver. 2.2.3^46^ and SAMtools ver. 0.1.19^47^. The number of fragments per kilobase of exon per million mapped fragments was calculated using Cufflinks ver. 2.2.143,44^48^ and visualized it as a heatmap. Pathway analyses were conducted using a STRING network tool. The raw data of this study are available under Gene Expression Omnibus (GEO) accession number GSE 175708.

### Cardiac magnetic resonance (CMR) imaging

Serial MRIs were conducted using a horizontal 7.0-T Bruker scanner (PharmaScan 70/16 USR, Bruker Biospin, Ettlingen, Germany). All MRI experiments were performed under general anaesthesia using 1%–2% isoflurane administered via a mask covering each animal’s nose and mouth. Respiratory signals, body temperature, and heart rate were monitored using a physiological monitoring system (SA Instruments, Calgary, Canada). Throughout all experiments, body temperatures were continuously maintained at 36.0 ± 0.5 °C by circulating water through heating pads^49^. The centre of each imaging slice was carefully positioned over the mouse heart. First, a 3-plane sequence was performed to define the slice orientation using self-gated cine imaging with navigator ech0 (IntraGate, Bruker). Next, 5 consecutive scans of the short axis from the apex to the base of the heart were obtained in 2- and 4-chamber long-axis views. These scans were used for fast low-angle shots with navigator echo using the following parameters: repetition time/echo time = 8.1/1.5 ms, flip angle = 15°, field of view = 2.56 × 2.56 cm, matrix = 192 × 192, slice thickness = 1.0 mm, number of repetitions = 250, number of concomitant slices covering the whole heart from the apex to base = 5, number of phases per cardiac cycle = 15, expected heart rate = 350 beats per minute (bpm), expected respiratory rate = 60 bpm, in-plane resolution per pixel = 142 µm, total acquisition time = 16 min 13 s, and total anaesthesia time = approximately 40 min.

### CMR image analysis

All CMR image analyses were performed retrospectively using cvi42 software (version 5.13.5. Circle Cardiovascular Imaging, Inc., Calgary, Canada). LV function and volumes were quantified from a stack of short-axis cine images. Papillary muscles were excluded from the LV volume. Circumferential strain and radial strain were analysed using a stack of short-axis cine images, whereas longitudinal strain was calculated from 2- and 4-chamber long-axis cine images. Epicardial and endocardial contours were manually drawn in the end-diastolic phase. Attenuation of each LV strain was depicted using a colour map based on strain values.

### Mouse hepatic stellate cells (HSCs) isolation

Mouse livers were perfused through the portal vein with solution I (137 mM NaCl, 5.4 mM KCl, 0.6 mM NaH_2_PO_4_, 0.8 mM Na2HPO4, 10 mM HEPES, 0.5 mM EGTA, 4.2 mM NaHCO3, 5 mM glucose, pH 7.4) for 5 min at 37 °C at a flow rate of 7 ml/minute. Each liver was then perfused with solution I containing 3.8 mM CaCl2 instead of EGTA (solution II) and 180 mg/l collagenase for 10 min at 37 °C. After perfusion, the liver was excised and incubated in solution II containing 400 mg/l pronase and 20 mg/l DNase at 37 °C for 20 min with gentle stirring. The incubation mixture was filtered through mesh (pore size: 150 μm). The filtrate was centrifuged at 450 g for 7 min. The fractions enriched with stellate cells and Kupffer/endothelial cells were then obtained by centrifugation with a triple-layered density cushion of Nycodenz (Gey’s balanced salt solution/8.2% Nycodenz/17% Nycodenz) at 1400 g for 20 min. Stellate cells in the upper white layer were washed by centrifugation at 450 g for 7 min, resuspended in DMEM supplemented with 10% FBS and antibiotics (105 U/l penicillin G, 100 mg/l streptomycin), and cultured on uncoated plastic plates (Falcon 3001, Thermo Fisher, CA, USA) at 37 °C in an incubator (5% CO2/95% air). A total of 3.75 × 10^6^ stellate cells were obtained from each mouse.

### Statistics

All results are presented as the mean ± SD, and p values were calculated using Student’s t test or Welch’s t test. One-way ANOVA with Tukey’s post hoc test was used to evaluate differences among 3 or more groups.

### Ethics approval and consent to participate

The experimental protocol was approved by the Ethics Review Committee for Animal Experimentation of Osaka University Graduate School of Medicine (28-056-024) and performed in conformity with ARRIVE guidelines.

## Supplementary Information

Supplementary Information.

## Data Availability

The datasets used and/or analysed during the current study are available from the corresponding author on reasonable request.

## Abbreviations

NAFLD: Nonalcoholic fatty liver disease
NASH: Nonalcoholic steatohepatitis
PPARα: Peroxisome proliferator-activated receptorα
H.E.: Haematoxylin–eosin
O.R.O.: Oil Red O
S.R.: Sirius Red
TC: Total cholesterol
FC: Free cholesterol
EC: Esterified cholesterol
TG: Triglycerides
NEFA: Nonesterified fatty acids
HDL: High density lipoprotein
LDL: Low density lipoprotein
MRI: Magnetic resonance imaging
MT: Masson trichrome
LV: Left ventricular
LVEF: Left ventricular ejection fraction
LVEDV: Left ventricular end-diastolic volume
LVESV: Left ventricular end-systolic volume
LVSV: Left ventricular stroke volume
LVM: Left ventricular mass
LS: Longitudinal strain
CS: Circumferential strain
RS: Radial strain
WGA: Wheat germ agglutinin
MW: Molecular weight
ROS: Reactive oxygen species
DHE: Dihydroethidium
DAPI: 4,6-Diamidino-2-phenylindole

## References

[1] Younossi ZM (2016). Global epidemiology of nonalcoholic fatty liver disease-Meta-analytic assessment of prevalence, incidence, and outcomes. Hepatology (Baltimore, Md.).

[2] Angulo P (2015). Liver fibrosis, but no other histologic features, is associated with long-term outcomes of patients with nonalcoholic fatty liver disease. Gastroenterology.

[3] Zhou YY (2018). Nonalcoholic fatty liver disease contributes to subclinical atherosclerosis: a systematic review and meta-analysis. Hepatol. Commun..

[4] Simon TG, Roelstraete B, Khalili H, Hagstrom H, Ludvigsson JF (2021). Mortality in biopsy-confirmed nonalcoholic fatty liver disease: results from a nationwide cohort. Gut.

[5] Nakahara T (2014). Type 2 diabetes mellitus is associated with the fibrosis severity in patients with nonalcoholic fatty liver disease in a large retrospective cohort of Japanese patients. J. Gastroenterol..

[6] Younossi Z (2018). Global burden of NAFLD and NASH: trends, predictions, risk factors and prevention. Nat. Rev. Gastroenterol. Hepatol..

[7] Lee YH (2018). Association of non-alcoholic steatohepatitis with subclinical myocardial dysfunction in non-cirrhotic patients. J. Hepatol..

[8] Canada JM (2019). Relation of hepatic fibrosis in nonalcoholic fatty liver disease to left ventricular diastolic function and exercise tolerance. Am. J. Cardiol.

[9] Nakamura H (2006). Primary prevention of cardiovascular disease with pravastatin in Japan (MEGA Study): a prospective randomised controlled trial. The Lancet.

[10] Suzuki K (2019). Elevated serum Non-HDL (high-density lipoprotein) cholesterol and triglyceride levels as residual risks for myocardial infarction recurrence under statin treatment. Arterioscler. Thromb. Vasc. Biol..

[11] A. S. , Group (2010). Effects of combination lipid therapy in type 2 diabetes mellitus. N. Engl. J. Med..

[12] Arai H (2017). Efficacy and safety of K-877, a novel selective peroxisome proliferator-activated receptor alpha modulator (SPPARMalpha), in combination with statin treatment: Two randomised, double-blind, placebo-controlled clinical trials in patients with dyslipidaemia. Atherosclerosis.

[13] Fruchart JC (2017). Pemafibrate (K-877), a novel selective peroxisome proliferator-activated receptor alpha modulator for management of atherogenic dyslipidaemia. Cardiovasc. Diabetol..

[14] Ida S, Kaneko R, Murata K (2019). Efficacy and safety of pemafibrate administration in patients with dyslipidemia: a systematic review and meta-analysis. Cardiovasc. Diabetol..

[15] Fruchart JC (2019). The selective peroxisome proliferator-activated receptor alpha modulator (SPPARMalpha) paradigm: conceptual framework and therapeutic potential : A consensus statement from the International Atherosclerosis Society (IAS) and the Residual Risk Reduction Initiative (R3i) Foundation. Cardiovasc. Diabetol..

[16] Fruchart JC, Hermans MP, Fruchart-Najib J, Kodama T (2021). Selective peroxisome proliferator-activated receptor alpha modulators (SPPARMalpha) in the metabolic syndrome: is pemafibrate light at the end of the tunnel?. Curr. Atheroscler. Rep..

[17] Pradhan AD (2018). Rationale and design of the pemafibrate to reduce cardiovascular outcomes by reducing triglycerides in patients with diabetes (PROMINENT) study. Am. Heart J..

[18] Araki M (2018). The peroxisome proliferator-activated receptor alpha (PPARalpha) agonist pemafibrate protects against diet-induced obesity in mice. Int. J. Mol. Sci..

[19] Honda Y (2017). Pemafibrate, a novel selective peroxisome proliferator-activated receptor alpha modulator, improves the pathogenesis in a rodent model of nonalcoholic steatohepatitis. Sci. Rep..

[20] Yoshida M (2020). Combination therapy with pemafibrate (K-877) and pitavastatin improves vascular endothelial dysfunction in dahl/salt-sensitive rats fed a high-salt and high-fat diet. Cardiovasc. Diabetol..

[21] Maruyama S (2002). Fenofibrate, a peroxisome proliferator-activated receptor alpha activator, suppresses experimental autoimmune myocarditis by stimulating the interleukin-10 pathway in rats. J. Atheroscler. Thromb..

[22] Kim BM, Abdelfattah AM, Vasan R, Fuchs BC, Choi MY (2018). Hepatic stellate cells secrete Ccl5 to induce hepatocyte steatosis. Sci. Rep..

[23] Takei K (2017). Effects of K-877, a novel selective PPARalpha modulator, on small intestine contribute to the amelioration of hyperlipidemia in low-density lipoprotein receptor knockout mice. J. Pharmacol. Sci..

[24] Shioi T (2002). Akt/protein kinase B promotes organ growth in transgenic mice. Mol. Cell Biol..

[25] Condorelli G (2002). Akt induces enhanced myocardial contractility and cell size in vivo in transgenic mice. Proc. Natl. Acad. Sci. U S A.

[26] Mantovani A (2019). Association between non-alcoholic fatty liver disease and risk of atrial fibrillation in adult individuals: an updated meta-analysis. Liver Int..

[27] Watanabe S (2018). A high-fat and high-cholesterol diet induces cardiac fibrosis, vascular endothelial, and left ventricular diastolic dysfunction in SHRSP5/Dmcr rats. J. Atheroscler. Thromb..

[28] Briand F (2021). Elafibranor improves diet-induced nonalcoholic steatohepatitis associated with heart failure with preserved ejection fraction in Golden Syrian hamsters. Metabol. Clin. Exp..

[29] Matsuzawa N (2007). Lipid-induced oxidative stress causes steatohepatitis in mice fed an atherogenic diet. Hepatology (Baltimore, Md.).

[30] Chang J (2020). Dietary oxysterol, 7-ketocholesterol accelerates hepatic lipid accumulation and macrophage infiltration in obese mice. Front. Endocrinol. (Lausanne).

[31] Kawaguchi M (2011). Inflammasome activation of cardiac fibroblasts is essential for myocardial ischemia/reperfusion injury. Circulation.

[32] Xiao H (2018). IL-18 cleavage triggers cardiac inflammation and fibrosis upon beta-adrenergic insult. Eur. Heart J..

[33] Sato M (2015). Fetuin-A negatively correlates with liver and vascular fibrosis in nonalcoholic fatty liver disease subjects. Liver Int. Off. J. Int. Assoc. Study Liver.

[34] Meex RCR, Watt MJ (2017). Hepatokines: linking nonalcoholic fatty liver disease and insulin resistance. Nat. Rev. Endocrinol..

[35] Kucukoglu O, Sowa JP, Mazzolini GD, Syn WK, Canbay A (2021). Hepatokines and adipokines in NASH-related hepatocellular carcinoma. J. Hepatol..

[36] Teratani T (2012). A high-cholesterol diet exacerbates liver fibrosis in mice via accumulation of free cholesterol in hepatic stellate cells. Gastroenterology.

[37] Montecucco F (2012). CC chemokine CCL5 plays a central role impacting infarct size and post-infarction heart failure in mice. Eur. Heart J..

[38] Takashima SKM (2007). HB-EGF, transactivation, and cardiac hypertrophy. Int. J. Gerontol..

[39] Tseng WY, Dou J, Reese TG, Wedeen VJ (2006). Imaging myocardial fiber disarray and intramural strain hypokinesis in hypertrophic cardiomyopathy with MRI. J. Magn. Reson. Imaging.

[40] Nakamori S (2018). Native T1 mapping and extracellular volume mapping for the assessment of diffuse myocardial fibrosis in dilated cardiomyopathy. JACC Cardiovasc. Imaging.

[41] Nakajima A (2021). Randomised clinical trial: Pemafibrate, a novel selective peroxisome proliferator-activated receptor alpha modulator (SPPARMalpha), versus placebo in patients with non-alcoholic fatty liver disease. Aliment. Pharmacol. Ther..

[42] Sairyo M (2018). A novel selective PPARalpha modulator (SPPARMalpha), K-877 (Pemafibrate), attenuates postprandial hypertriglyceridemia in mice. J. Atheroscler. Thromb..

[43] Delerive P (1999). Peroxisome proliferator-activated receptor alpha negatively regulates the vascular inflammatory gene response by negative cross-talk with transcription factors NF-kappaB and AP-1. J. Biol. Chem..

[44] Kleiner DE (2005). Design and validation of a histological scoring system for nonalcoholic fatty liver disease. Hepatology (Baltimore Md.).

[45] Kim D (2013). TopHat2: accurate alignment of transcriptomes in the presence of insertions, deletions and gene fusions. Genome Biol..

[46] Langmead B, Salzberg SL (2012). Fast gapped-read alignment with Bowtie 2. Nat. Methods.

[47] Bonfield JK (2021). HTSlib: C library for reading/writing high-throughput sequencing data. Gigascience.

[48] Trapnell C (2010). Transcript assembly and quantification by RNA-Seq reveals unannotated transcripts and isoform switching during cell differentiation. Nat. Biotechnol..

[49] Saito S, Takahashi Y, Ohki A, Shintani Y, Higuchi T (2019). Early detection of elevated lactate levels in a mitochondrial disease model using chemical exchange saturation transfer (CEST) and magnetic resonance spectroscopy (MRS) at 7T-MRI. Radiol. Phys. Technol..

