# Supplementary material for: Pemafibrate suppresses NLRP3 inflammasome activation in the liver and heart in a novel mouse model of steatohepatitis-related cardiomyopathy
Source: Sci Rep. 2022 Feb 22;12:2996. doi: 10.1038/s41598-022-06542-8 (PMC8863801; doi:10.1038/s41598-022-06542-8)
Supplement: Supplementary file 1 — Supplementary Information. [file 41598_2022_6542_MOESM1_ESM.pdf]

Supplementary Information

Pemafibrate suppresses NLRP3 inflammasome activation in the liver and heart in a novel mouse model of steatohepatitis-related cardiomyopathy

Kanno K. et al.

Supplemental Table S1. Taqman® primers used for real-time quantitative PCR.

Probe	Cat. Number
Tumour necrosis factor a (Tnfa)	Mm00443258_m1
Interleukin 1 beta (Il1b)	Mm00434228_m1
Interleukin 6 (Il6)	Mm00446190_m1
Transforming growth factor beta (Tgfb)	Mm01178820_m1
Collagen type I alpha 1 chain (Col1a1)	Mm00801666_m1
Collagen type I alpha 2 chain (Col1a2)	Mm00483888_m1
3-Hydroxy-3-Methylglutaryl-CoA synthase 1 (Hmgcs1)	Mm01304569_m1
3-hydroxy-3-methyl-glutaryl-CoA reductase (Hmgr)	Mm01282499_m1
Acetyl-CoA carboxylase 1 (Acc1)	Mm01304257_m1
Fatty acid synthase (Fasn)	Mm00662319_m1
Stearoyl-CoA desaturase-1 (Scd1)	Mm00772290_m1
Uncoupling protein 3 (Ucp3)	Mm01163394_m1
Carnitine palmitoyltransferase I a (Cpt1a)	Mm01231183_m1
Acyl-CoA oxidase 1 (Acox)	Mm01246835_m1
Chemokine (C-C motif) ligand 5 (Ccl5)	Mm01302427_m1
Chemokine (C-C motif) ligand 2 (Ccl2, MCP-1)	Mm00441242_m1
Matrix metallopeptidase 9 (MMP9)	Mm00442991_m1
Nucleotide-binding oligomerization domain-like receptor family, pyrin domain-containing 3 (Nlrp3)	Mm00840904_m1
Liver X receptors α (LXRα)	Mm00443451_m1
ATP-binding cassette transporter A1 (Abca1)	Mm00442646_m1
Sterol regulatory element-binding transcription factor 1 (Srebf1)	Mm00550338_m1
Sterol regulatory element-binding transcription factor 2 (Srebf2)	Mm01306292_m1
Fibroblast growth factor 21 (Fgf21)	Mm00840165_g1
Uncoupling protein-2 (Ucp2)	Mm00627599_m1
Microsomal triglyceride transfer protein (Mttp)	Mm00435015_m1
Caspase-1 (Casp1)	Mm00438023_m1
Carnitine palmitoyltransferase 1b (Cpt1b)	Mm00487200_m1
RNA polymerase II subunit A (Polr2a)	Mm00839493_m1
Beta-actin (β-actin)	Mm00607939_s1

Supplemental Table S2. Primary antibodies for western blotting.

Antigen	Cat. Number
Caspase-1 (Casp1)	14-9832-82
Asc	AL177
Protein kinase B (Akt)	2920S
Phospho-protein kinase B (pAkt)	4060S
Mechanistic target of rapamycin (mTOR)	2972S
Phospho-mechanistic target of rapamycin (pmTOR)	2971S
Glyceraldehyde-3-phosphate dehydrogenase (Gapdh)	8884
Liver X receptors α (LXRα)	Ab176323
ATP-binding cassette transporter A1 (Abca1)	NB400-105
Sterol regulatory element-binding protein 1 (Srebf1)	Sc-366
Sterol regulatory element-binding protein 2 (Srebf2)	Ab30682
Microsomal triglyceride transfer protein (Mtp)	Ab75316
CD36	Ab133625
LC3	2775S
Rubicon	8465S
p62	23214S
Becklin-1	3738S
β-actin	A3854

Supplemental Table S3. Fluorescent conjugated antibodies for flow cytometry analysis.

Antibody	Clone
FITC anti-Ly6G	1A8 (BioLegend)
PerCP/Cyanine5.5 anti-CD45	30-F11 (BioLegend)
APC anti-CD3e	145-2C11 (BioLegend)
APC-Cy7 anti-CD11b	M1/70 (BioLegend)
PE anti-NK1.1	PK136 (BioLegend)
PE-Cy7 anti-CD19	6D5 (BioLegend)

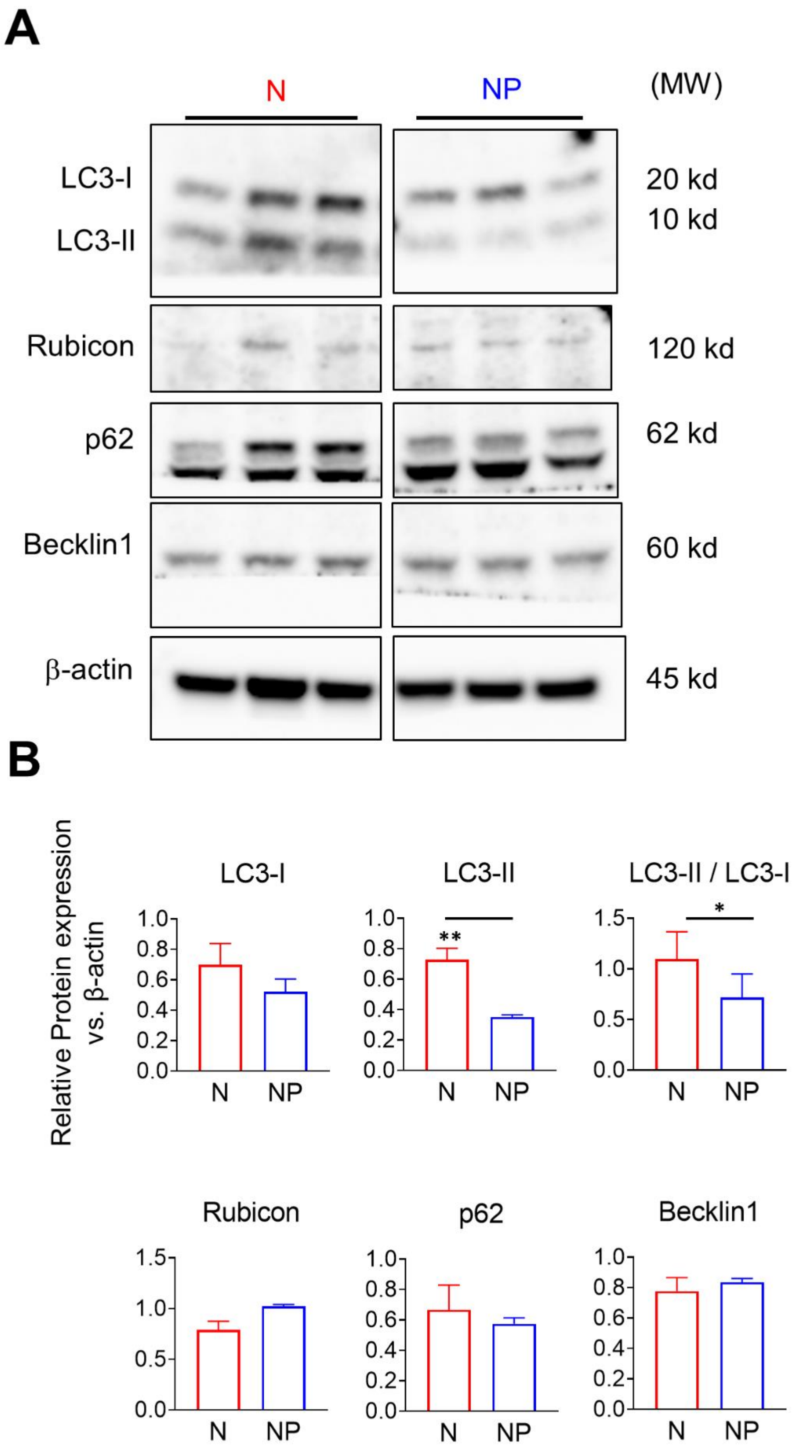

Supplemental Figure 1. Autophagy function of the liver. (A) and (B) Liver western blots for LC3-I, LC3-II, Rubicon, p62, Becklin1, and β-actin. Full-length blots are presented in Supplementary Figure X. Images were scanned and quantified by ImageJ software. The results are presented as the mean ± SD, and p values were calculated using Student's t test. ** $p<0.01$, N vs. NP group. MW, molecular weight

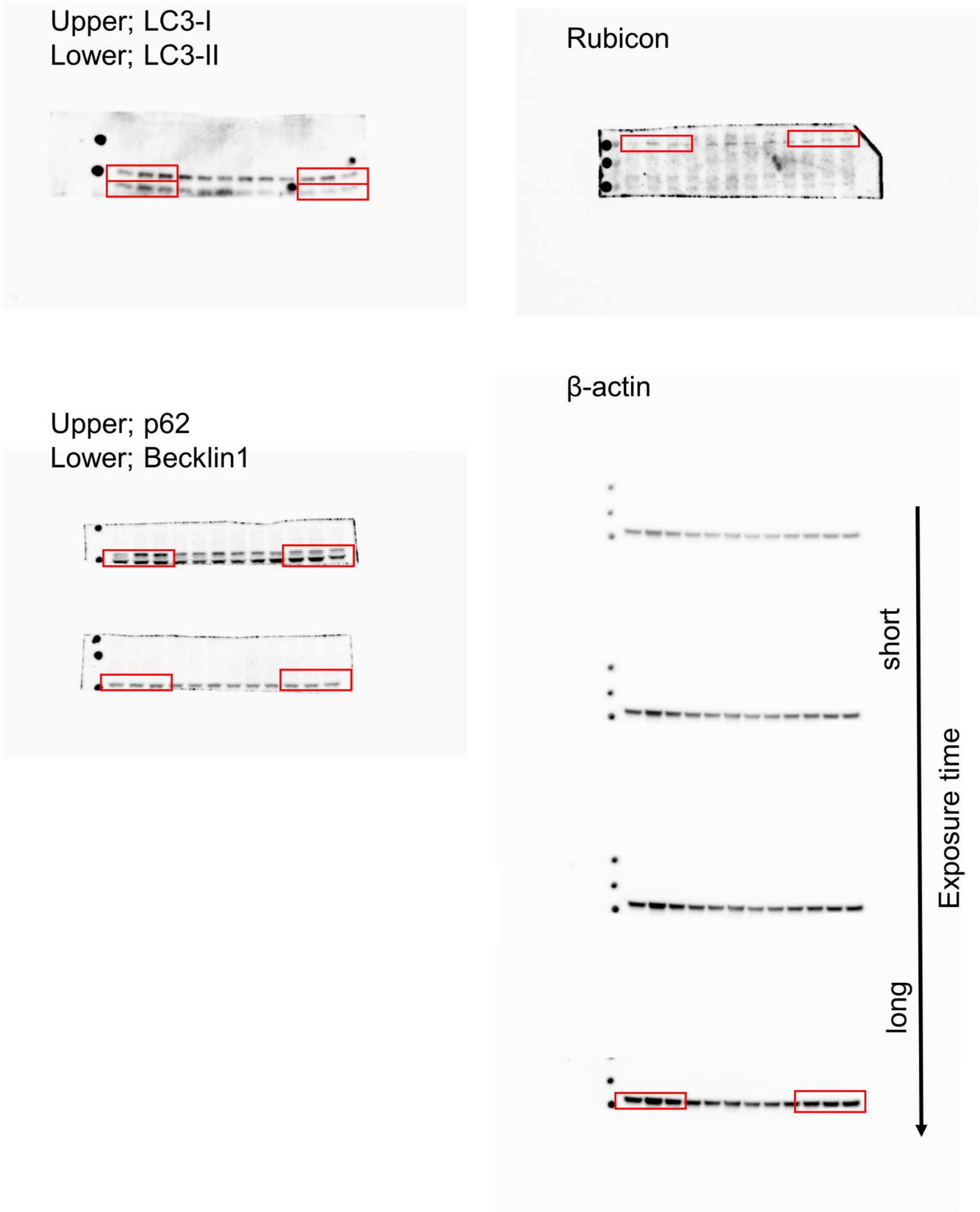

Supplemental Figure 2. Full-length blots/gels of proteins of the liver presented in Supplemental Figure 1.

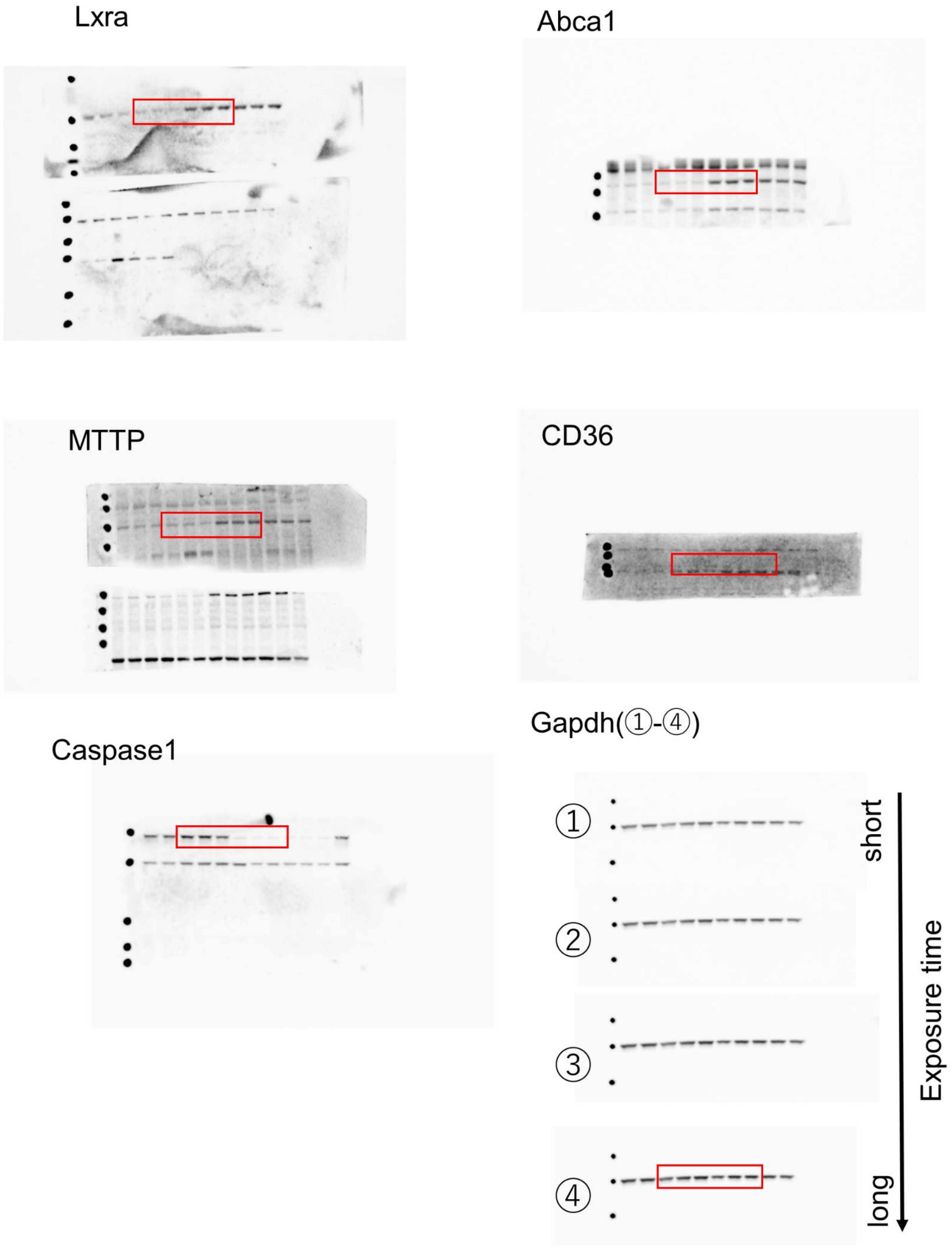

Supplemental Figure 3. Full-length blots/gels of proteins of the liver presented in Figure 2E.

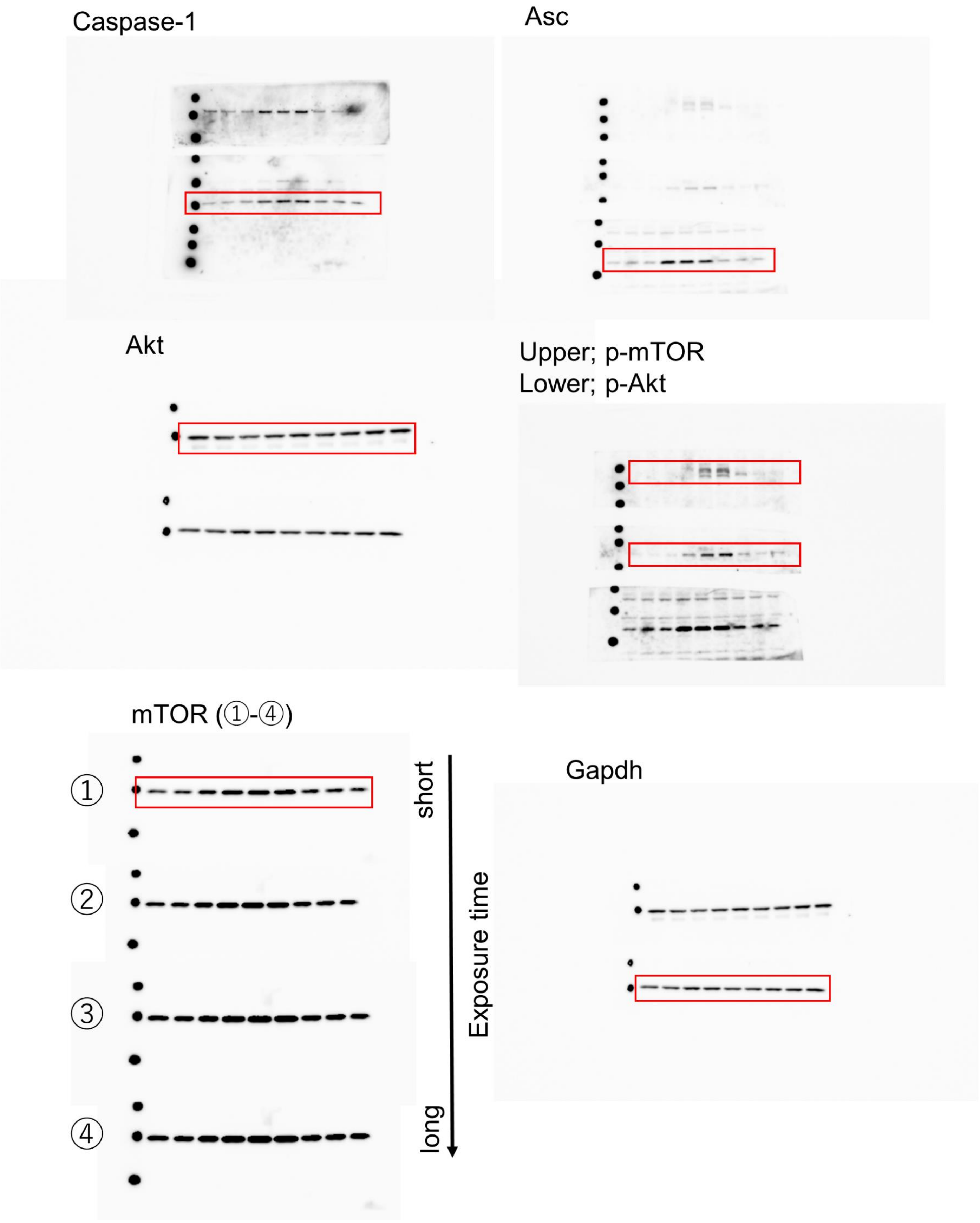

Supplemental Figure 4. Full-length blots/gels of proteins of the heart presented in Figure 5B.

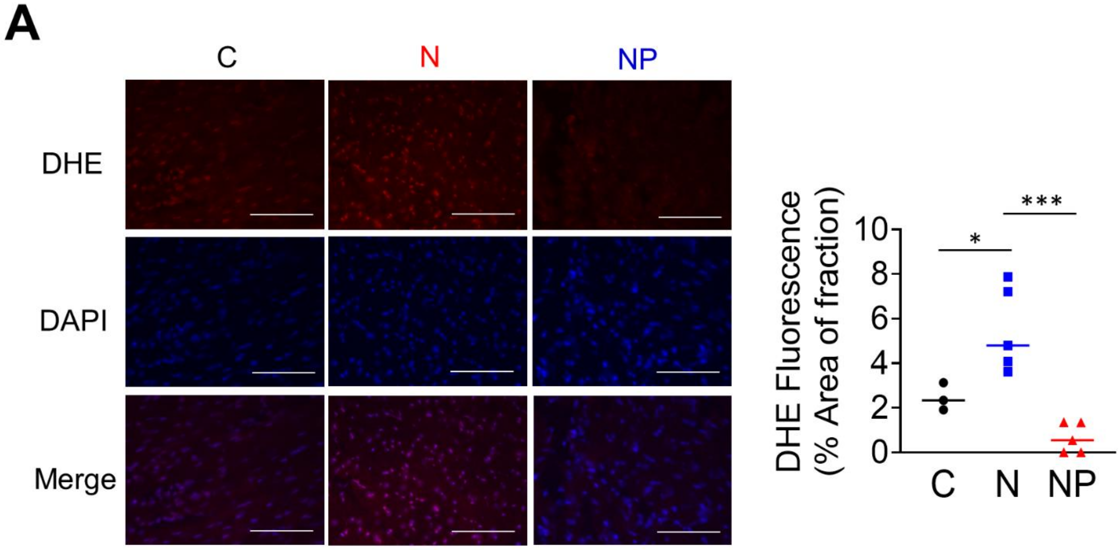

Supplemental Figure 5. ROS production in myocardium. (A) Reactive oxygen species in the myocardium were measured by fluorescence using dihydroethidium. The results are presented as the mean ± SD, and p values were calculated using one-way ANOVA with Tukey's post hoc test.
* $p<0.05$ and *** $p<0.001$, C vs. N group or N vs. NP group. The number of mice in each group: C, n=3; N, n=5; NP, n=5. ROS, reactive oxygen species; DHE, dihydroethidium; DAPI, 4,6-diamidino-2-phenylindole

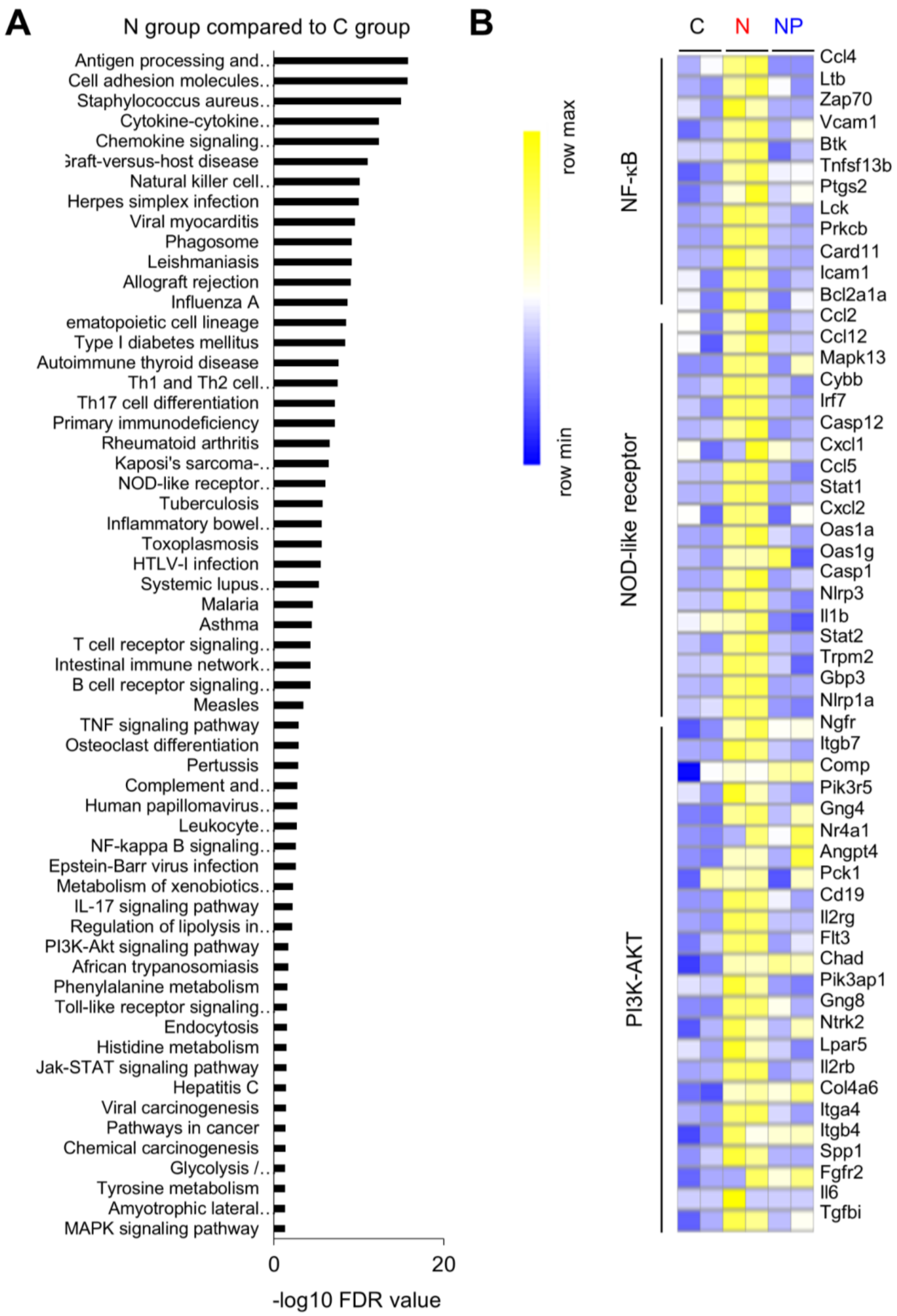

Supplemental Figure 6. Pathway analysis and heatmap analysis of the heart created based on RNA sequencing. (A) Pathways upregulated in the N group compared to the C group. (B) Heatmap imaging of genes related to the NF-κB, NOD-like receptor, and PI3K-Akt signalling pathways. The number of mice in each group: C, n=2; N, n=2; NP, n=2. FDR, false discovery rate. Statistical analyses were carried out using FDR correction. A default FDR<0.05 was considered statistically significant.
